# Estimation after blinded sample size reassessment

**DOI:** 10.1177/0962280216670424

**Published:** 2016-10-02

**Authors:** Martin Posch, Florian Klinglmueller, Franz König, Frank Miller

**Affiliations:** 1 Section for Medical Statistics, Center for Medical Statistics, Informatics, and Intelligent Systems, Medical University of Vienna, Vienna, Austria; 2 Department of Statistical Sciences, University of Padua, Padua, Italy; 3 Department of Statistics, Stockholm University, Stockholm, Sweden Martin Posch and Florian Klinglmueller share first authorship.

**Keywords:** Adaptive design, interim analysis, internal pilot study, point estimate, sample size reassessment

## Abstract

Blinded sample size reassessment is a popular means to control the power in clinical trials if no reliable information on nuisance parameters is available in the planning phase. We investigate how sample size reassessment based on blinded interim data affects the properties of point estimates and confidence intervals for parallel group superiority trials comparing the means of a normal endpoint. We evaluate the properties of two standard reassessment rules that are based on the sample size formula of the *z*-test, derive the worst case reassessment rule that maximizes the absolute mean bias and obtain an upper bound for the mean bias of the treatment effect estimate.

## 1 Introduction

In clinical trials for the comparison of means of normally distributed observations, the sample size to achieve a specific target power depends on the true effect size and variance. For the purpose of sample size planning, the effect size is usually assumed to be equal to a minimally clinically relevant effect while the variance is often estimated from historical data. For situations where only little prior knowledge on the variance is available, clinical trial designs with sample size reassessment based on interim estimates of this nuisance parameter have been proposed. Stein^1^ developed a two-stage procedure, where the second stage sample size is calculated based on a first stage variance estimate aiming to achieve a pre-specified target power. In the context of clinical trials, several extensions of Steins two-stage procedure have been considered^2–8^ that all require an unblinding of the interim data for the computation of the variance estimate. However, regulatory agencies generally prefer blinded interim analyses as they entail less potential for bias.^9–13^

Gould and Shih^14^ proposed to estimate the variance from the blinded interim data by computing the variance from the total sample (pooling the observations from both groups), instead. Although this interim estimate is not consistent^15^ and has a positive bias if the alternative hypothesis holds, the bias is negligible for effect sizes typically observed in clinical trials.^16^ Furthermore, sample size reassessment based on the total variance has no relevant impact on the type I error rate in parallel group superiority trials^17^ (see also the literature^18–21^) and achieves the target power well. Similar results on sample size reassessment based on blinded nuisance parameter estimates were obtained for binary data,^22^ count data^23–25^ longitudinal data,^26^ and for fully sequential sample size reassessment.^27^ For non-inferiority trials with normal endpoints, a minor inflation of the type I error rate for small sample sizes has been observed.^28,29^ However, if the sample size reassessment rule is not only based on the primary endpoint but also on blinded secondary or safety endpoint data, the type I error rate for the test of the primary endpoint may be substantially inflated.^30^

While for adaptive clinical trials with sample size reassessment based on the unblinded interim treatment effect estimate, it is well known that unadjusted point estimates of the effect size and confidence intervals may be biased;^31,32^ the properties of point estimates and confidence intervals computed at the end of an adaptive clinical trial with blinded sample size reassessment have received less attention. In this paper, we investigate the bias and standard error of the treatment effect point estimate and compare the absolute mean bias due to blinded sample size reassessment to upper boundaries derived for adaptive designs with unblinded sample size reassessment.^33^ We also investigate the bias of the final variance estimate under sample size reassessment rules that are based on the blinded interim variance estimate. We also investigate the bias of the final variance estimate under sample size reassessment rules that are based on the blinded interim variance estimate. Previously, this bias had been investigated for a corresponding unblinded reassessment rule (which had no upper sample size bound), sharp bounds for the bias had been derived and an additive bias correction had been suggested.^8^ Here, in this paper, we derive corresponding bounds for the bias of the final variance estimate in the blinded case. Furthermore, we assess the coverage probability of one- and two-sided confidence intervals.

In Section 2, we introduce adaptive designs with blinded sample size reassessment. Theoretical results on the bias of estimates are presented in Section 3. In Section 4, we report a simulation study quantifying the bias and coverage probabilities in a variety of scenarios. The impact of the results is discussed in the context of a case study in Section 5. Section 6 concludes the paper with a discussion and recommendations. Technical proofs are given in Appendix 1.

## 2 Blinded sample size reassessment

Consider a parallel group comparison of the means $\mu_{a},\mu_{b}$ of a normally distributed endpoint with common unknown variance $\sigma^{2}$. The one-sided null hypothesis $H_{0}:\delta \leq 0$, is tested against $H_{1}:\delta >0$ at level *α*, where $\delta =\mu_{b}-\mu_{a}$ denotes the true effect size. Let $\delta_{0}$ denotes the alternative for which the trial is powered and assume that in the planning phase a first stage per group sample size $n_{1}\geq 2$ is chosen based on an a priori variance estimate $\sigma_{0}^{2}$. Note, however, that all results below depend only on the chosen *n*_1_ and not the way it has been determined. As a consequence, for given *n*_1_, they also apply if it has been determined with other justifications. After the endpoints of the first stage subjects are observed, in a blinded interim analysis, the one-sample variance estimate is computed, given by

$$S_{1,\mathrm{OS}}^{2}=\frac{1}{2n_{1}-1}[\sum_{i=a,b}\sum_{k=1}^{n_{1}}(X_{i1k}-{\bar{X}}_{\cdot 1\cdot }{)}^{2}]$$

where *X_ijk_* is the observation $k=1,\ldots ,n_{j}$ in stage *j* = 1, 2 for treatment i=a,b and ${\bar{X}}_{\cdot 1\cdot }$ is the mean of the pooled first stage samples. Based on $S_{1,\mathrm{OS}}^{2}$, the second stage sample size is chosen with a pre-specified sample size function $n_{2}(S_{1,\mathrm{OS}}^{2})$. Below we drop the argument of the function *n*_2_ for convenience if the meaning is clear from the context. Then, the overall sample size is $n=n_{1}+n_{2}$ per group. After the study is completed and unblinded, we assume that the point estimates for the mean difference and the variance

$$\bar{\Delta }={\bar{X}}_{b\cdot \cdot }-{\bar{X}}_{a\cdot \cdot }S^{2}=\frac{1}{2n-2}[\sum_{i=a,b}\sum_{j=1}^{2}\sum_{k=1}^{n_{j}}(X_{\mathrm{ijk}}-{\bar{X}}_{i\cdot \cdot }{)}^{2}]$$

are computed, where ${\bar{X}}_{i\cdot \cdot }=\sum_{j=1}^{2}\sum_{k=1}^{n_{j}}X_{\mathrm{ijk}}/n,i=a,b$. Furthermore, (i) the standard fixed sample lower confidence bound corresponding to the one-sided null hypothesis *H*_0_ given by $\bar{\Delta }-t_{2n-2,1-\alpha }S\sqrt{2/n}$, where $t_{2n-2,1-\alpha }$ denotes the 1-α quantile of the central *t*-distribution with 2n-2 degrees of freedom, (ii) the upper confidence bound—corresponding to the complementary null hypotheses $H_{0}^{-}:\delta \geq 0$—given by $\bar{\Delta }+t_{2n-2,1-\alpha }S\sqrt{2/n}$ and (iii) the two-sided confidence interval given by $\bar{\Delta }\pm t_{2n-2,1-\alpha /2}S\sqrt{2/n}$ is determined.

As example consider a sample size reassessment rule aiming to control the power under a pre-specified absolute treatment effect. It is derived from the standard sample size formula for the comparison of means of normally distributed observations with a one-sided *z*-test using the variance estimate $S_{1,\mathrm{OS}}^{2}$

(1) $$n_{2}^{u}(S_{1,\mathrm{OS}}^{2})=\min\{n_{2\max},\max\{n_{2\min},2(z_{1-\alpha }+z_{1-\beta }{)}^{2}\frac{S_{1,\mathrm{OS}}^{2}}{\delta_{0}^{2}}-n_{1}+1\}\}$$

where 1-β is the desired power and $n_{2\min}$ and $n_{2\max}$ are pre-specified minimal and maximal second stage sample sizes with $0\leq n_{2\min}<n_{2\max}$. The +1 in formula (1) was added for mathematical convenience to derive a lower bound for the variance estimate in Theorem 4 below. Given that we anyway have to round the sample size to an integer, this modification is of minor practical importance. While $S_{1,\mathrm{OS}}^{2}$ is an unbiased variance estimator for $\sigma^{2}$ when the true effect size is zero, it is positively biased by $\delta^{2}n_{1}/(4n_{1}-2)$ for effect sizes δ¬=0.^5^ A sample size reassessment rule that is based on an adjusted variance estimate which is unbiased under the effect size *δ*_0_ for which the trial is powered is obtained from equation (1) by replacing $S_{1,\mathrm{OS}}^{2}$ by $S_{1,\mathrm{OS}}^{2}-\delta_{0}^{2}n_{1}/(4n_{1}-2)$ and is given by

(2) $$n_{2}^{a}(S_{1,\mathrm{OS}}^{2})=\min\{n_{2\max},\max\{n_{2\min},2(z_{1-\alpha }+z_{1-\beta }{)}^{2}(\frac{S_{1,\mathrm{OS}}^{2}}{\delta_{0}^{2}}-\frac{n_{1}}{4n_{1}-2})-n_{1}+1\}\}$$

## 3 Theoretical results on the bias of the final mean and variance estimators

In this section, we consider adaptive two-stage designs with general sample size reassessment rules where the second stage sample size is given as some non-constant function $n_{2}:{\mathbb{R}}^{+}\to {\mathbb{N}}_{0}$ of the blinded interim variance estimate $S_{1,\mathrm{OS}}^{2}$. We derive the bias of the final effect size and variance estimators $\bar{\Delta }$ and *S*^2^.

**Theorem 1**

Consider an adaptive two-stage design with a general sample size reassessment rule $n_{2}(S_{1,\mathrm{OS}}^{2})$.

- The bias $E(\bar{\Delta }-\delta )$ is given by
  $$\begin{array}{c} \int_{0}^{\infty }\int_{0}^{\infty }\frac{t(2n_{1}-2)(r(q,\delta -t)-r(q,\delta +t))}{\sigma^{2}[1+r(q,\delta +t)][1+r(q,\delta -t)]}\times \\ \phi_{0,\sqrt{2\sigma^{2}/n_{1}}}(t)\chi_{2n_{1}-2}^{2}(\frac{q(2n_{1}-2)}{\sigma^{2}})\text{d}t\text{d}q \end{array}$$

where

$$r(x,y)=n_{2}(\frac{2(n_{1}-1)}{2n_{1}-1}x+\frac{n_{1}}{2(2n_{1}-1)}y^{2})/n_{1}$$

- Under the null hypothesis *δ* = 0, the final mean estimator $\bar{\Delta }$ is unbiased.
- If $n_{2}(S_{1,\mathrm{OS}}^{2})$ is increasing and δ>0 (δ<0) the estimator $\bar{\Delta }$ is negatively (positively) biased, respectively. Furthermore, the absolute bias is symmetric in *δ* around 0.
- If the sample size reassessment rule is increasing and $\lim_{S_{1,\mathrm{OS}}^{2}\to \infty }n_{2}(S_{1,\mathrm{OS}}^{2})=\infty$, the bias of $\bar{\Delta }$ converges to 0 for δ→±∞.

Note that the sample size functions $n_{2}^{u},n_{2}^{a}$ are increasing such that the property (c) applies. If $n_{2\max}=\infty$, $n_{2}^{u},n_{2}^{a}$ tend to infinity for $S_{1,\mathrm{OS}}^{2}\to \infty$ such that the property (d) applies.

In Theorem 2, we give an upper bound for the bias of the mean in adaptive two-stage designs with a general sample size reassessment rule $n_{2}(S_{1,\mathrm{OS}}^{2})$ such that $n_{2\min}\leq n_{2}(S_{1,\mathrm{OS}}^{2})\leq n_{2\max}$, for lower and upper bounds where $n_{2\min},n_{2\max}$, where $n_{2\max}$ may be infinite (corresponding to the case of unrestricted sample size reassessment). For general unblinded sample size reassessment rules that may depend on the fully unblinded interim data, the upper bound for the bias is^33^

(3) $$(\frac{1}{n_{1}+n_{2\min}}-\frac{1}{n_{1}+n_{2\max}})0.4\sigma \sqrt{2n_{1}}$$

and is realized for the sample size reassessment rule that sets $n_{2}=n_{2\min}$ if ${\bar{\Delta }}_{1}\leq \delta$ and $n_{2}=n_{2\max}$ otherwise. This upper bound obviously also applies for the case of blinded sample size reassessment based on the total variance estimate because the set of unblinded sample size reassessment rules for which *n*_2_ may depend on the unblinded interim data in any way, includes all blinded sample size reassessment rules. This bound is not sharp if the sample size depends on $S_{1,\mathrm{OS}}^{2}$, only. In the theorem below, we derive the sample size reassessment rule that maximizes the bias and compute the corresponding maximal bias which is a sharp upper bound for the bias that can occur under any sample reassessment based on sample size rules $n_{2}(S_{1,\mathrm{OS}}^{2})$. We show that for increasing effect sizes, the bias approaches the bias of unblinded sample size reassessment.

**Theorem 2**

Consider an adaptive two-stage design with a general sample size reassessment rule $n_{2}(S_{1,\mathrm{OS}}^{2})$, such that $0\leq n_{2\min}\leq n_{2}(S_{1,\mathrm{OS}}^{2})\leq n_{2\max}$, where $n_{2\max}$ may be infinite. Then,

- the expected first stage treatment effect estimate conditional on the variance estimate $S_{1,\mathrm{OS}}^{2}$ is given by
  $$\begin{array}{c} E({\bar{\Delta }}_{1}|S_{1,\mathrm{OS}}^{2})=\int_{-b}^{b}x\phi_{\delta ,\sqrt{2\sigma^{2}/n_{1}}}(x)\\ \times \chi_{2n_{1}-2}^{2}\{[(2n_{1}-1)S_{1,\mathrm{OS}}^{2}-x^{2}n_{1}/2]/\sigma^{2}\}\mathrm{dx}/K \end{array}$$

where ${\bar{\Delta }}_{1}$ denotes the unblinded first stage estimate of the mean treatment effect, and $b=\sqrt{2S_{1,\mathrm{OS}}^{2}(2n_{1}-1)/n_{1}},K=\int_{-b}^{b}\phi_{\delta ,\sqrt{2\sigma^{2}/n_{1}}}(x)\chi_{2n_{1}-2}^{2}\{[(2n_{1}-1)S_{1,\mathrm{OS}}^{2}-x^{2}n_{1}/2]/\sigma^{2}\}\mathrm{dx}$

- the bias of $\bar{\Delta }$ is maximized for the sample size rule
  (4) $$\begin{array}{c} n_{2}(S_{1,\mathrm{OS}}^{2})=\{\begin{array}{cc} n_{2\min} & \text{ if }E({\bar{\Delta }}_{1}|S_{1,\mathrm{OS}}^{2})>\delta \\ n_{2\max} & \text{ otherwise } \end{array} \end{array}$$
- the maximum bias of $\bar{\Delta }$ depends on the true effect size *δ* and is given by
  (5) $$(\frac{n_{1}}{n_{1}+n_{2\min}}-\frac{n_{1}}{n_{1}+n_{2\max}})E(E({\bar{\Delta }}_{1}-\delta |S_{1,\mathrm{OS}}^{2})1_{\left\{E({\bar{\Delta }}_{1}|S_{1,\mathrm{OS}}^{2})>\delta \right\}})$$
- For δ→∞ and σ fixed, the maximum bias of $\bar{\Delta }$ caused by blinded sample size reassessment rules based on $S_{1,\mathrm{OS}}^{2}$ converges to the maximum bias caused by unblinded sample size reassessment rules given by equation (3).

Note that, by symmetry, the *negative* bias of $\bar{\Delta }$ is maximized by the sample size rule equation (4) and is given by equation (5) with the inequality signs reversed. If $n_{2\min}=0$ and $n_{2\max}=\infty$, the first factor in equation (5) reduces to 1.

In Theorem 3, we derive the bias of the variance estimator computed at the end of the trial for general sample size reassessment rules $n_{2}(S_{1,\mathrm{OS}}^{2})$ and show that it is symmetric in the true treatment effect *δ* and converges to 0 for increasing *δ* under suitable conditions (which are satisfied for $n_{2}^{u},n_{2}^{a}$ if $n_{2\max}=\infty$).

**Theorem 3**

Consider an adaptive two-stage design with a general sample size reassessment rule $n_{2}(S_{1,\mathrm{OS}}^{2})$. Then,

- the bias of the variance estimator *S*^2^ is given by
  $$\begin{array}{c} \int_{0}^{\infty }\int_{-\infty }^{\infty }\frac{1}{\left(n_{1}-1+n_{1}r(q,t+\delta )\right)}((n_{1}-1)(q-\sigma^{2})+\frac{t^{2}n_{1}/2-\sigma^{2}}{2+2r(q,t+\delta )})\\ \times \phi_{0,\sqrt{2\sigma^{2}/n_{1}}}(t)\chi_{2n_{1}-2}^{2}(q\gamma )\gamma \text{d}t\text{d}q \end{array}$$

where $\gamma =(2n_{1}-2)/\sigma^{2}$ and *r* is defined in Theorem 1;

- the bias of *S*^2^ is symmetric in *δ* around 0;
- if $\lim_{S_{1,\mathrm{OS}}^{2}\to \infty }n_{2}(S_{1,\mathrm{OS}}^{2})=\infty$, the bias of *S*^2^ converges to 0 for δ→±∞.

In Theorem 4, we derive a lower bound for the bias of the variance estimator *S*^2^ (which is negative) in an adaptive two-stage design with sample size reassessment rule $n_{2}^{u}$.

**Theorem 4**

Consider a design with blinded sample size reassessment based on $S_{1,\mathrm{OS}}^{2}$, where the second stage sample size is set to $n_{2}^{u}(S_{1,\mathrm{OS}}^{2})$. Under the null hypothesis *δ* = 0, the bias of the variance estimator *S*^2^ at the end of the study has the following bounds

$$-\frac{2n_{1}-1}{2n_{1}-3}\cdot \frac{1}{v}<{\mathrm{ES}}^{2}-\sigma^{2}<0$$

where $v=2(z_{1-\alpha }+z_{1-\beta }{)}^{2}/\delta_{0}^{2}$. If $n_{2\max}=\infty$ (no upper restriction for the sample size), the bias converges to the lower bound for large variances

$${\mathrm{ES}}^{2}-\sigma^{2}\to -\frac{2n_{1}-1}{2n_{1}-3}\cdot \frac{1}{v}\text{ for }\sigma^{2}\to +\infty$$

Note that this lower bound has a similar form as the bound derived for the bias in adaptive two-stage designs with sample size reassessment based on the unblinded variance estimate.^8^ In the latter setting, the bound is given by $-(n_{1}-1)/[(n_{1}-2)v]$. The bound derived in Theorem 4 does not apply to designs where the adjusted rule $n_{2}^{a}$ is applied. The numerical results below suggest that for the adjusted rule the absolute bias is maximized for a finite $\sigma^{2}$ and not for $\sigma^{2}\to \infty$ as for the unadjusted rule.

## 4 Simulation study of the properties of point estimates and confidence intervals

**Figure 1.**
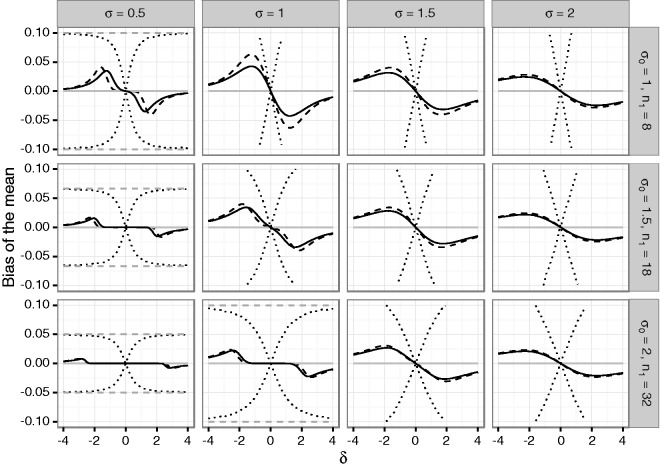
Bias of the mean under blinded sample size reassessment using the unadjusted (solid line) and adjusted (dashed line) interim variance estimate. The dotted lines show maximum negative and positive bias that can be attained under any blinded sample size reassessment rule according to Theorem 2. The dashed gray line shows the maximum bias that can be attained under any (unblinded) sample size reassessment rule. The treatment effect used for planning is set to $\delta_{0}=1$. Rows refer to the a priori assumed standard deviations *σ*_0_ determining the first stage sample size *n*_1_. The columns correspond to actual standard deviations. The *x*-axis in each graph denotes the true treatment effect *δ*, the *y*-axis shows the bias.

**Figure 2.**
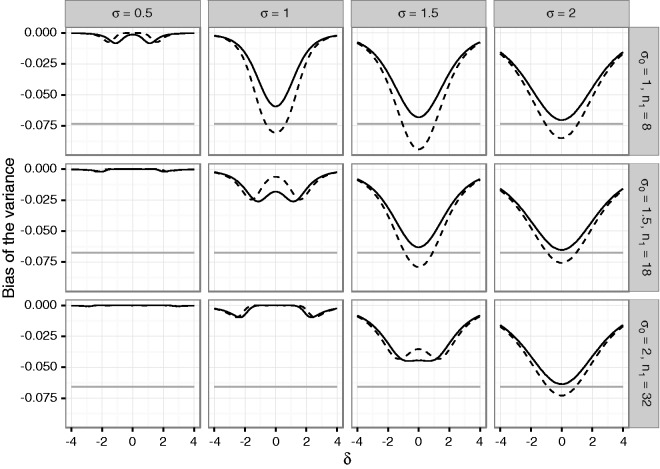
Bias of the variance under blinded sample size reassessment using the unadjusted (solid line) and adjusted (dashed line) interim variance estimate. The gray line gives the lower bound from Theorem 4 for the bias under sample size reassessment based on the unadjusted variance estimate. The treatment effect used for planning is set to $\delta_{0}=1$. Rows refer to the a priori assumed standard deviations *σ*_0_ determining the first stage sample size *n*_1_. The columns correspond to actual standard deviations. The *x*-axis in each graph denotes the true treatment effect *δ*, the *y*-axis shows the bias.

To quantify the bias of mean and variance estimates as well as the actual coverage of corresponding one- and two-sided confidence intervals in adaptive two-stage designs with blinded sample size reassessment, a simulation study was performed. We assumed that a trial is planned for an effect size $\delta_{0}=1$ and a priori variance estimate *σ*_0_ at a one-sided significance level α=0.025. The preplanned sample size of a two-armed parallel group trial was chosen to provide 80% power (based on the normal approximation) for $\sigma_{0}\in \{1,1.5,2\}$. The first stage sample size was set to half of the preplanned total sample size leading to first stage per group sample sizes of $n_{1}\in \{8,18,32\}$. For each first stage sample size, first stage data were simulated from normal distributions for effect sizes *δ* ranging from −2 to 2 (in steps of 0.1) and common standard deviations *σ*, ranging from 0.5 to 2 in steps of 0.5. The second stage sample sizes were then reassessed to $n_{2}^{u},n_{2}^{a}$ (with $n_{2\min}=0$). Below we refer to the latter reassessment rules as the unadjusted and adjusted sample size reassessment rules. For each scenario $5\times 10^{7}$, trials were simulated with R.^34^ The source code is available in the R-package blindConfidence which can be downloaded and installed from github. Details on the implementation and additional results are presented in the Supplementary Material. Figures 1 and 2 show the mean bias of the final estimates of the mean and variance based on the total sample. If the alternative holds, the estimates may be substantially biased for small first stage sample sizes.

For both sample size reassessment rules, the bias of the mean is zero under the null hypothesis and has the opposite sign as the true effect size, otherwise (in accordance with Theorem 1). For very large positive and negative effect sizes, the bias is close to zero. This is due to the fact that a large effect size results in a large positive bias of the blinded interim variance estimates which in turn results in very large second stage sample sizes. As a consequence, the overall estimates are essentially equal to the (unbiased) second stage estimates, and the bias becomes negligible. In the considered scenarios, the absolute bias is decreasing in the first stage sample size. This also holds for the upper bound of the bias for general sample size reassessment rules. However, while the bias for the adjusted and unadjusted reassessment rules is small for larger sample sizes, the upper bound for general sample size reassessment rules still exceeds 10% in the considered scenarios even in the case where $n_{1}=32$ subjects per group are recruited in the first stage. In the considered scenarios, we observe that the upper bound for the bias of the mean estimate increases in the true effect size *δ* and the true standard deviation *σ*. The numerical results confirm that for increasing |δ| the maximum bias under blinded sample size reassessment approaches to the maximum bias under unblinded sample size reassessment as shown in Theorem 2.

The variance estimate is negatively biased for both considered sample size reassessment rules. For increasing variances, the absolute bias under the null hypothesis *δ* = 0 for the rule $n_{2}^{u}$ approaches the lower bound derived in Theorem 4. For large positive and negative effect sizes, the bias approaches zero, again, because the overall estimate becomes essentially equal to the (unbiased) second stage estimates. In general, we observe that using the adjusted interim variance estimate for sample size reassessment results in a larger absolute bias of mean and variance estimates.

**Figure 3.**
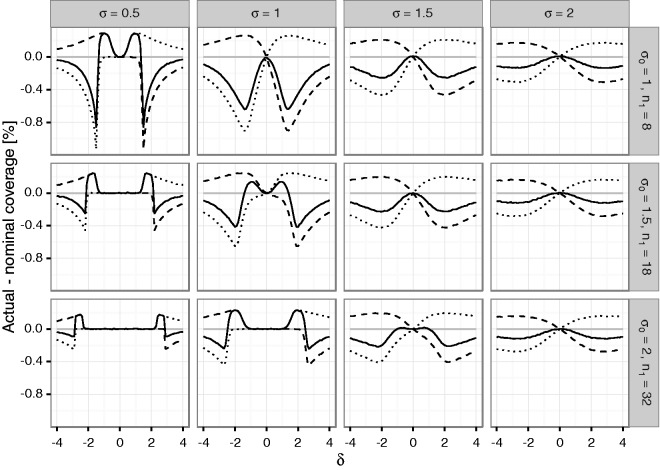
Difference between actual and nominal coverage probabilities in percentage points under blinded sample size reassessment using the *unadjusted interim variance estimate* $S_{1,\mathrm{OS}}$: upper confidence bound (dashed line), lower confidence bound (dotted line) and two-sided interval (solid line). Rows refer to the a priori assumed standard deviations *σ*_0_ determining the first stage sample size *n*_1_. The columns correspond to actual standard deviations. The *x*-axis in each graph denotes the true treatment effect *δ*; the *y*-axis shows the difference of actual to nominal coverage probability such that negative values indicate settings where the confidence bound is not valid.

The difference of the coverage probabilities of the confidence intervals to the nominal coverage probabilities (i.e., 0.975 for the one-sided, 0.95 for the two-sided confidence interval) is shown in Figure 3 for the unadjusted sample size reassessment rule (see Figure 1 of the Supplementary Material for corresponding results for the adjusted sample size reassessment rule). For positive *δ*, the lower confidence bound is conservative (with coverage probability larger than 0.975) while the upper bound is anti-conservative, and vice-versa for negative *δ*. In fact, due to symmetry, the coverage probability for the upper confidence bound at a certain true effect size *δ* is the same as the coverage probability of the lower confidence bound at -δ. If the true $\sigma^{2}$ and the true |δ| are small, the trial will stop with minimal sample size almost with probability 1. Then, sample size is essentially fixed, no bias occurs and coverage is as desired. This is, e.g., the case for $\sigma =1,n_{1}=32$ and |δ|<1.3 and explains the exact coverage there. Similar cases occur for small |δ| in the three pictures for σ=0.5 as well.

The two-sided coverage probability (which is given by one minus the sum of the non-coverage probabilities of the lower and upper bounds) is not controlled over a large range of *δ*. However, for *δ* = 0 (i.e., under the null hypothesis), the inflation of the non-coverage probability, which corresponds to the Type I error rate of the corresponding test, is minor for small sample sizes (0.5 to 0.01 percentage points $n_{1}<10$) and essentially controlled for larger samples sizes. (See Figure 15 of the Supplementary Material for a simulation study that aims to identify the *σ* where the Type I error rate of the original hypothesis test is maximized.) The good coverage under the null hypothesis is at first sight surprising, given that the variance estimate is negatively biased and the mean estimate is unbiased. We therefore computed the actual variance $\sigma_{e}^{2}$ of the mean estimate in the simulation study. To compare the actual variance of the mean to the estimated variance, we computed for each simulated trial the variance estimate S^e2=2S2n1+n2 as well as the actual variance $\sigma_{f}^{2}=\frac{2\sigma^{2}}{n_{1}+n_{2}}$ of a design with fixed sample sizes *n*_1_, *n*_2_ (thus, ignoring that *n*_2_ is dependent on the first stage sample). Both quantities were then averaged over the Monte-Carlo samples. The results show that the true variance of the mean estimate of an adaptive design with blinded sample size reassessment is smaller than the average variance of a fixed sample design with the same sample sizes. The bias of the estimate of the variance of the mean estimate is close to zero around the null hypothesis. This holds both for designs using the adjusted and unadjusted interim variance estimates to reassess the sample size and gives some intuition why there is no relevant inflation of the coverage probability of confidence intervals under the null hypotheses, even though the variance estimate has a considerable negative bias (see Figures 5 and 6 of the Supplementary Material for results that show the variance of the mean estimate for designs that use the unadjusted and adjusted sample size rule).

**Figure 4.**
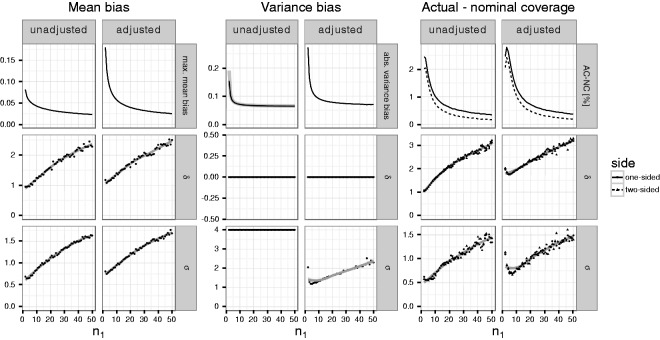
Maximum absolute mean and variance bias as well as maximum negative difference between actual and nominal coverage probabilities (in percentage points) for given per group first stage sample sizes *n*_1_ between 2 and 50; values of *δ* between 0 and 4; *σ* between 0.5 and 4. Left columns show the results for the unadjusted sample size reassessment rule, right columns for the adjusted sample size reassessment rule. The first row shows the value for bias and non-coverage, the second shows the effect size *δ* and the third shows the standard deviation *σ* at which the specific value is attained. The gray line denotes a Loess smoothed estimate. For the maximum absolute variance bias, based on the unadjusted rule, the theoretical bound is shown using the slightly thicker gray line.

While Figures 1 to 3 demonstrate the dependence of the bias and coverage probabilities on the true mean and standard deviation, in an actual trial these parameters are typically unknown. Therefore, we computed the maximum absolute bias of the mean, variance and the maximum difference between nominal and actual coverage probabilities for fixed first stage sample sizes over a range of values for the true mean and variance (see Figure 4). The optimization was performed in several steps based on simulations across an increasingly finer grid of true mean differences δ∈[0,4] and standard deviations σ∈[.5,4] and using increasingly larger numbers of up to 10^8^ simulation runs (see Section 2 of the Supplementary Material for computational details of this simulation).

Overall, the maximum bias of the mean and variance estimates as well as the maximum difference between nominal and actual coverage probabilities using the adjusted sample size reassessment rule exceed that of the unadjusted rule. The maximum absolute bias of the mean bias (Columns 1 and 2 of Figure 4) drops from 0.18 (0.08) for trials with a per group first stage sample size of $n_{1}=2$ to 0.02 (0.02) for trials with a per group first stage sample size of $n_{1}=50$ if the adjusted (unadjusted) interim variance estimate is used to reassess the sample size. For increasing values of the first stage sample size, the maximum of the absolute mean bias is found for larger values of the true mean and variance.

For the unadjusted rule, the simulations suggest that the absolute variance bias is maximized for *δ* = 0 and σ→∞ (see Figure 2 and the additional simulations in Section 2 of the Supplementary Material). Thus, Column 3 of Figure 4 shows the absolute bias for *δ* = 0 and *σ* = 4 (the largest value of *σ* considered in the parameter grid). For the adjusted rule, Column 4 of Figure 4 shows the maximum absolute bias for *δ* = 0 and *σ* maximized over the grid. For the adjusted rule, the maximum bias is attained for a finite *σ*. The maximum absolute bias of the variance estimate drops from 0.27 (0.19) for trials with a per group first stage sample size of $n_{1}=2$ to 0.07 (0.06) for trials with a per group first stage sample size of $n_{1}=50$ if the adjusted (unadjusted) interim variance estimate is used to reassess the sample size. In the simulation, the maxima of the absolute variance bias, using the unadjusted reassessment rule, is practically identical to the theoretical boundary derived in Theorem 4 (see the gray line in the first graph of Column 3 of Figure 4). If the adjusted rule is used, the maximum is attained for values of *σ* ranging from 1 to 2, increasing with the first stage sample size.

The maximum negative difference between actual and nominal coverage probabilities (“AC-NC,” in Columns 5 and 6 of Figure 4) of the one-sided confidence intervals ranges from about 2.8 (2.4) to 0.4 (0.3) percentage points if the adjusted (unadjusted) variance estimate is used to reassess the sample size. For the two-sided confidence intervals, differences between actual and nominal coverage are slightly lower ranging from 2.4 (2) to 0.2 (0.2) percentage points, respectively. For increasing first stage sample sizes, the maximum is attained for increasing values of both *σ* and *δ* where the former ranges between 1 and 3 and the latter between 0.5 and 1.5.

In Section 4 of the Supplementary Material, the corresponding results for restricted sample size rules that limit the maximum second stage sample size to twice the preplanned second stage sample size, are given. We observe that for scenarios where $\sigma_{0}<\sigma$, limiting the second stage sample size results in a reduction of the (absolute) bias and higher coverage probabilities. This, however, comes at the price of limited control in terms of power. When $\sigma_{0}>\sigma$ we observe that the (absolute) bias of the mean estimate (in line with the theoretical results in Theorem 2) is reduced by a factor $n_{2\max}/(n_{1}+n_{2\max})$. The bias of the variance estimate as well as the difference between actual and nominal coverage probabilities are less affected by restricting the second stage sample size. When *σ* is substantially smaller than *σ*_0_ bias and coverage probabilities under the restricted and unrestricted sample size reassessment rules are nearly identical.

## 5 Case study

We illustrate the estimation of treatment effects with a randomized placebo controlled trial to demonstrate the efficacy of the kava-kava special extract “WS® 1490” for the treatment of anxiety^35^ that was used to illustrate procedures for blinded sample size adjustment.^17^ The primary endpoint of the study was the change in the Hamilton Anxiety Scale (HAMA) between baseline and end of treatment. Assuming a mean difference between treatment and control of $\delta_{0}=5.5$ and a standard deviation of $\sigma_{0}=8$ (i.e., a variance of $\sigma_{0}^{2}=64$) results in a sample size of 34 patients per group to provide a power of 80%.

As in the previous case study,^17^ we assume that sample size reassessment based on blinded data is performed after 15 patients per group, that the sample size reassessment rules were applied with $n_{2\min}=0$ and $n_{2\max}=\infty$ and consider that the interim estimate of the standard deviation is $S_{1,\mathrm{OS}}=6$. Consequently, we get $n_{2}^{u}=4.7$ and $n_{2}^{a}=0.6$, such that in the second stage five patients per group would have been recruited based on the unadjusted sample size reassessment rule $n_{2}^{u}$, and only one patient per group based the adjusted rule $n_{2}^{a}$.

**Figure 5.**
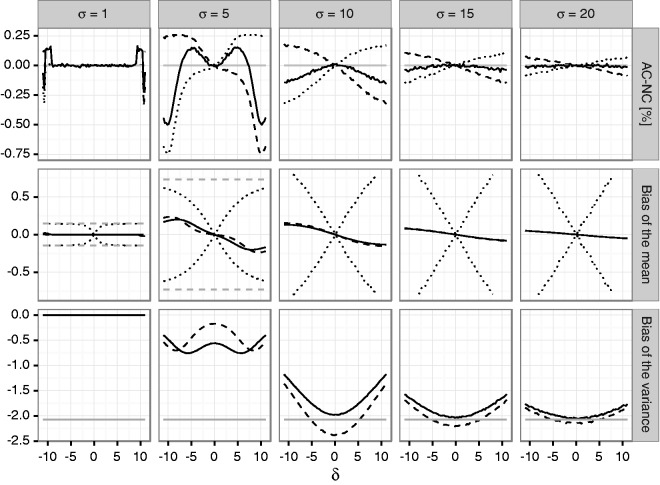
Coverage probabilities and bias of mean and variance estimates for the case study. The first row of panels shows actual-nominal coverage probabilities (AC-NC) for the 97.5% upper (dashed line), lower (dotted line) and the 95% two-sided confidence intervals, for the unadjusted reassessment rule $n_{2}^{u}$. The second row shows the bias of the mean estimate if $n_{2}^{u}$ (solid line) or $n_{2}^{a}$ (dashed line) is used. The dotted line shows upper and lower bounds for the bias for general blinded sample size reassessment rules based on $S_{1,\mathrm{OS}}^{2}$. The dashed gray line shows the bounds for the bias for a general unblinded sample size reassessment rule. The third panel shows the bias of the variance estimate if either $n_{2}^{u}$ (solid line) or $n_{2}^{a}$ (dashed line) is used. The red line shows the theoretical boundary for the bias give in Theorem 4.

To estimate the bias of the mean and variance estimate as well as the coverage probabilities of confidence intervals, we performed a simulation study for true effect sizes *δ* ranging from −11 $\left(=-2\delta_{0}\right)$ to 11 $\left(=2\delta_{0}\right)$ in steps of 0.05, and *σ* from 1 to 20 in steps of 1 (see Figure 5).

### 5.1 Unadjusted sample size rule

For fixed *δ*, the absolute bias of the variance estimate is increasing in *σ*. For *σ* = 20, the bias of the variance becomes -2.06 which is within simulation error of the theoretical lower bound. The absolute mean bias takes its maximum (minimum) 0.2 (−0.2) for the effect sizes δ=-7.98 (7.98), respectively, and a standard deviation of σ=5. The maximum inflation of the non-coverage probabilities of one-sided 97.5% and two-sided 95% confidence intervals is 0.7 and 0.5 percentage points, respectively. The actual coverage probabilities are smallest for large absolute values of the true mean difference and standard deviations of around 5.

### 5.2 Adjusted sample size rule

If the sample size reassessment is based on the adjusted variance estimate, the absolute bias of the variance and mean estimate will be even larger taking values up to 2.49 for the variance and up to 0.25 for the mean, respectively. The inflation of the non-coverage probabilities goes up to 0.9 percentage points for the one-sided confidence intervals and 0.6 percentage points for the two-sided intervals.

## 6 Discussion

We investigated the properties of point estimates and confidence intervals in adaptive two-stage clinical trials where the sample size is reassessed based on blinded interim variance estimates. Such adaptive designs are in accordance with current regulatory guidance that proposes blinded sample size reassessment procedures based on aggregated interim data.^9–11^ We showed that such blinded sample size reassessment may lead to biased effect size estimates, biased variance estimates and may have an impact on the coverage probability of confidence intervals. The extent of the biases depends on the specific sample size reassessment rule, the first stage sample size, the true effect size and the variance.

We showed that for the unadjusted and the adjusted sample size reassessment rules that aim to control the power at the pre-specified level, the bias of confidence intervals may be large for very small first stage sample sizes but is small otherwise. Under the null hypothesis, even for first stage sample sizes as low as 16, the confidence intervals do not exhibit a relevant inflation of the non-coverage probability. For positive effect sizes, inflations (even though minor) are observed also for somewhat larger sample sizes. This corresponds to previous findings that the type I error rate of superiority as well as non-inferiority tests is hardly affected by blinded sample size reassessment.^17,28^ In addition, we show that for positive treatment effects the lower confidence interval (which is often the most relevant because it gives a lower bound of the treatment effect) is strictly conservative in all considered simulation scenarios. The upper bound in contrast shows an inflation of the non-coverage probability, which is however only of relevant size if sample sizes are small.

An approach to obtain conservative confidence intervals even for trials with small first stage sample sizes is to apply methods proposed for flexible designs with unblinded interim analyses^32,33,36–39^ which are based on combination tests or the conditional error rate principle. Such approaches allow to construct confidence intervals which control the coverage probabilities. However, they are based on test statistics that do not equally weigh outcomes from patients recruited in the first and second stage. A hybrid approach that strictly controls the coverage probability could be based on confidence intervals that are the union of the fixed sample confidence interval and the confidence interval obtained from the flexible design methodology. Similar approaches have been proposed for hypothesis testing in adaptive designs.^40,41^

We demonstrated that the treatment effect estimates are negatively (positively) biased under the adjusted and unadjusted sample size reassessment rules for all positive (negative) effect sizes. The bias decreases with the first stage sample size but for worst case parameter constellations it remains noticeable also for larger sample sizes. In addition, for the worst case sample size reassessment rule based on blinded variance estimates, the bias may be substantial even for large first stage sample sizes: As the effect size increases the maximum bias of the blinded sample size reassessment rule approaches the bias of unblinded sample size reassessment. As a consequence to maintain the integrity of confirmatory clinical trials with blinded sample size reassessment, binding sample size adaptation rules must be pre-specified and it is important to verify on a case-by-case basis that the reassessment rules used do not have a substantial impact on the properties of estimators should be used.

## Supplementary Material

Supplementary material

## Supplementary material

Supplementary figures and additional details on the simulation study are presented in an additional document, which is distributed with the paper as Supplementary Material.

The R code used to compute the results of the simulation, and the case study is available as an R-package named blindConfidence which is available on github or on request from the authors.

## Appendix 1: Proofs of the Theorems

Note that the blinded first stage variance estimate can be written as^17^

(6) $$S_{1,\mathrm{OS}}^{2}=\frac{2(n_{1}-1)}{2n_{1}-1}S_{1}^{2}+\frac{n_{1}}{2(2n_{1}-1)}{\bar{\Delta }}_{1}^{2}$$

where ${\bar{\Delta }}_{1}$ denotes the unblinded first stage estimates of the mean treatment effect and $S_{1}^{2}$ the (unblinded) pooled variance estimate. Thus, every second stage sample size rule $n_{2}(S_{1,\mathrm{OS}}^{2})$ can be written as function $n_{2}(S_{1}^{2},{\bar{\Delta }}_{1})$ of $S_{1}^{2}$ and ${\bar{\Delta }}_{1}$ which is symmetric in ${\bar{\Delta }}_{1}$ around 0. We can then write $r(x,y)=n_{2}(x,y)/n_{1}.$ Below we switch between both parameterizations of *n*_2_.

### Proof of Theorem 1

Proof (a) Let the true effect size *δ* be fixed. The bias of $\bar{\Delta }$ is given by $\mathrm{Cov}(\frac{1}{1+r(S_{1}^{2},{\bar{\Delta }}_{1})},{\bar{\Delta }}_{1})$, see Brannath et al.,^33^ which can be computed by

(7) $$\begin{array}{c} E(\frac{{\bar{\Delta }}_{1}}{1+r(S_{1}^{2},{\bar{\Delta }}_{1})})-E(\frac{1}{1+r(S_{1}^{2},{\bar{\Delta }}_{1})})E({\bar{\Delta }}_{1})=E(\frac{{\bar{\Delta }}_{1}-\delta }{1+r(S_{1}^{2},{\bar{\Delta }}_{1})})\\ =\int_{0}^{\infty }\int_{-\infty }^{\infty }\frac{t}{1+r(q,\delta +t)}\phi_{0,\sqrt{2\sigma^{2}/n_{1}}}(t)\chi_{2n_{1}-2}^{2}(q\gamma )\gamma \text{d}t\text{d}q\\ =\int_{0}^{\infty }\int_{0}^{\infty }t\frac{r(q,\delta -t)-r(q,\delta +t)}{\left[1+r(q,\delta +t)][1+r(q,\delta -t)\right]}\phi_{0,\sqrt{2\sigma^{2}/n_{1}}}(t)\chi_{2n_{1}-2}^{2}(q\gamma )\gamma \text{d}t\text{d}q \end{array}$$

where $\gamma =(2n_{1}-2)/\sigma^{2}$. (b) and (c) The sign of the integral is determined by the sign of $n_{2}(q,t+\delta )-n_{2}(q,-t+\delta )$. Note that *r*(*q*, *y*) is symmetric in *y* around 0 and increasing for *y* > 0 since this is true for $n_{2}(q,y)$. Thus, the integral is zero for *δ* = 0, negative for positive *δ* and positive for negative *δ*. Also the symmetry of the absolute bias follows by the symmetry of $n_{2}(q,y)$ in *y* around 0. (d) The first factor in equation (7) tends to 0, and the proof follows with the dominated convergence theorem. □

### Proof of Theorem 2

Proof (a) By equation (6), the joint density of ${\bar{\Delta }}_{1}$ and $S_{1,\mathrm{OS}}^{2}$ is given by

$$h(x,v)=\phi_{\delta ,\sqrt{2\sigma^{2}/n_{1}}}(x)\chi_{2n_{1}-2}^{2}\{[(2n_{1}-1)v-x^{2}n_{1}/2]/\sigma^{2}\}\cdot (2n_{1}-1)/\sigma^{2}$$

for v≥0 and |x|≤b and h(x,v)=0 otherwise. Therefore, the conditional distribution of ${\bar{\Delta }}_{1}$ given $S_{1,\mathrm{OS}}^{2}=v$ is $h(x,v)/\int_{-b}^{b}h(x,v)\mathrm{dx}$ and (a) follows.

(b) Note that the bias is given by

(8) $$\begin{array}{c} E(\bar{\Delta }-\delta )=E[({\bar{\Delta }}_{1}-\delta )\frac{n_{1}}{n_{1}+n_{2}(S_{1,\mathrm{OS}}^{2})}]\\ =E\{E[({\bar{\Delta }}_{1}-\delta )\frac{n_{1}}{n_{1}+n_{2}(S_{1,\mathrm{OS}}^{2})}|S_{1,\mathrm{OS}}^{2}]\} \end{array}$$

(9) $$=E\{E[E(({\bar{\Delta }}_{1}-\delta )|S_{1,\mathrm{OS}}^{2})\frac{n_{1}}{n_{1}+n_{2}(S_{1,\mathrm{OS}}^{2})}|S_{1,\mathrm{OS}}^{2}]\}$$

For fixed $S_{1,\mathrm{OS}}^{2}$, the term within the outer conditional expectation in equation (8) is deterministic and decreases in *n*_2_ if $E({\bar{\Delta }}_{1}|S_{1,\mathrm{OS}}^{2})>\delta$ and increases if $E({\bar{\Delta }}_{1}|S_{1,\mathrm{OS}}^{2})<\delta$. Therefore, the sample size rule equation (4) maximizes the bias.

(c) We plug-in the worst case sample size rule equation (4) into equation (9) and obtain

$$\begin{array}{c} E(\bar{\Delta }-\delta )=E[\frac{n_{1}}{n_{1}+n_{2\min}}E({\bar{\Delta }}_{1}-\delta |S_{1,\mathrm{OS}}^{2})1_{\left\{E({\bar{\Delta }}_{1}|S_{1,\mathrm{OS}}^{2})>\delta \right\}}]\\ +E[\frac{n_{1}}{n_{1}+n_{2\max}}E({\bar{\Delta }}_{1}-\delta |S_{1,\mathrm{OS}}^{2})1_{\left\{E({\bar{\Delta }}_{1}|S_{1,\mathrm{OS}}^{2})\leq \delta \right\}}] \end{array}$$

Because ${\bar{\Delta }}_{1}$ is unbiased such that $E({\bar{\Delta }}_{1}-\delta |S_{1,\mathrm{OS}}^{2})1_{\left\{E({\bar{\Delta }}_{1}|S_{1,\mathrm{OS}}^{2})>\delta \right\}}=-E({\bar{\Delta }}_{1}-\delta |S_{1,\mathrm{OS}}^{2})1_{\left\{E({\bar{\Delta }}_{1}|S_{1,\mathrm{OS}}^{2})\leq \delta \right\}}$ we get equation (5).

(d) Let N denotes a set of sample size reassessment rules $n_{2,\delta }(S_{1,\mathrm{OS}}^{2},{\bar{\Delta }}_{1})$ that may depend on $\delta ,S_{1,\mathrm{OS}}^{2},{\bar{\Delta }}_{1}$. Given a sample size rule $n_{2,\delta }(\cdot )\in N$, the bias can be written as

(10) $$\begin{array}{c} E_{\delta }(\bar{\Delta }-\delta )=E_{\delta }(({\bar{\Delta }}_{1}-\delta )\frac{n_{1}}{n_{1}+n_{2,\delta }(S_{1,\mathrm{OS}}^{2},{\bar{\Delta }}_{1})})\\ =\int_{-\infty }^{\infty }\int_{0}^{\infty }y\frac{n_{1}}{n_{1}+n_{2,\delta }(v,y+\delta )}\phi_{0,\sigma /\sqrt{n_{1}}}(y)\\ \times \chi_{2n_{1}-2}^{2}\{[(2n_{1}-1)v-(y+\delta {)}^{2}n_{1}/2]/\sigma^{2}\}\text{d}v\text{d}y \end{array}$$

The set of integrands of the outer integral in equation (10) for second stage sample size rules in N is uniformly integrable because the fraction $n_{1}/[n_{1}+n_{2,\delta }(y+\delta ,v)]\leq 1$ and the $\chi^{2}$-density is bounded (since $n_{1}\geq 2$).

The *unblinded* sample size reassessment rule that maximizes the bias for given *δ* is^33^

$$\begin{array}{c} n_{2,\delta }({\bar{\Delta }}_{1})=\{\begin{array}{cc} n_{2\min} & \text{ if }{\bar{\Delta }}_{1}>\delta \\ n_{2\max} & \text{ otherwise } \end{array} \end{array}$$

Consider the blinded sample size rules

$$\begin{array}{c} {\overset{∼}{n}}_{2,\delta }(S_{1,\mathrm{OS}}^{2})=\{\begin{array}{cc} n_{2\min} & \text{ if }S_{1,\mathrm{OS}}^{2}>\delta^{2}\frac{n_{1}}{2(2n_{1}-1)}\\ n_{2\max} & \text{ otherwise } \end{array} \end{array}$$

We show that for fixed *σ* and δ→∞ the second stage sample sizes of the blinded sample size rule ${\overset{∼}{n}}_{2}(S_{1,\mathrm{OS}}^{2})\to n_{2}({\bar{\Delta }}_{1})$, almost surely. Because, as shown above, the corresponding integrands in equation (10) are uniformly integrable, and it follows that also the respective biases converge and (d) follows.

Let ε>0. Then, there exists an $\varepsilon_{1},1/3>\varepsilon_{1}>0$, such that for all *δ* we have $P_{\delta }(|{\bar{\Delta }}_{1}-\delta |>3\varepsilon_{1})>1-\varepsilon$. Furthermore, there exists a $\delta_{0}>2$ such that for all $\delta >\delta_{0}$ we have $P_{\delta }(S_{1}^{2}/\delta <\varepsilon_{1})>1-\varepsilon$ and $P_{\delta }({\bar{\Delta }}_{1}>0)>1-\varepsilon$. Thus, for the event $A=\{{\bar{\Delta }}_{1}>0\}\cap \{S_{1}^{2}/\delta <\varepsilon_{1}\}\cap \{|{\bar{\Delta }}_{1}-\delta |>3\varepsilon_{1}\}$ we have $P_{\delta }(A)\geq 1-3\varepsilon$. However, on the set *A* we have $n_{2,\delta }({\bar{\Delta }}_{1})={\overset{∼}{n}}_{2,\delta }(S_{1,\mathrm{OS}}^{2})$: If $n_{2,\delta }({\bar{\Delta }}_{1})=n_{2\min}$ then ${\bar{\Delta }}_{1}>\delta$ and on *A* also ${\bar{\Delta }}_{1}>\delta +3\varepsilon_{1}$. Therefore, by equation (6)

$$S_{1,\mathrm{OS}}^{2}>\delta^{2}(1+6\varepsilon_{1}/\delta +9\varepsilon_{1}^{2}/\delta^{2})\frac{n_{1}}{2(2n_{1}-1)}>\delta^{2}\frac{n_{1}}{2(2n_{1}-1)}$$

and ${\overset{∼}{n}}_{2,\delta }(S_{1,\mathrm{OS}}^{2})=n_{2\min}$. On the other hand, if $n_{2,\delta }({\bar{\Delta }}_{1})=n_{2\max}$ then ${\bar{\Delta }}_{1}<\delta$ and on *A* also $0\leq {\bar{\Delta }}_{1}<\delta -3\varepsilon_{1}$. Therefore, by equation (6), because $2(n_{1}-1)/(2n_{1}-1)\leq 1$ and $1/3\geq n_{1}/2(2n_{1}-1)\geq 1/4$ and on *A* we have $S_{1}^{2}\leq \varepsilon_{1}\delta$

$$\begin{array}{c} S_{1,\mathrm{OS}}^{2}<\varepsilon_{1}\delta +\frac{n_{1}}{2(2n_{1}-1)}(\delta^{2}-6\varepsilon_{1}\delta +9\varepsilon_{1}^{2})\\ \leq \delta^{2}\frac{n_{1}}{2(2n_{1}-1)}+\varepsilon_{1}\delta -\frac{3}{2}\varepsilon_{1}\delta +3\varepsilon_{1}^{2}\\ \leq \delta^{2}\frac{n_{1}}{2(2n_{1}-1)}-\frac{\varepsilon_{1}\delta }{2}+3\varepsilon_{1}^{2}\leq \delta^{2}\frac{n_{1}}{2(2n_{1}-1)} \end{array}$$

where the last inequality follows because δ>2 and $\varepsilon_{1}<1/3$. □

### Proof of Theorem 3

Proof Let ${\bar{\Delta }}_{j}={\bar{X}}_{\mathrm{aj}\cdot }-{\bar{X}}_{\mathrm{bj}\cdot }$ be the observed mean difference between the treatments in stage *j* = 1 and (if $n_{2}>0$) in stage *j* = 2.

We consider first the case that $n_{2}>0$; the following considerations are under the condition of $S_{1}^{2}$ and ${\bar{\Delta }}_{1}$ such that they lead to $n_{2}>0$. Let $\Omega_{i}={\bar{X}}_{i1\cdot }-{\bar{X}}_{i2\cdot }$ be the observed mean difference between the stages for treatment i=a,b. Let further $S_{2}^{2}$ be the (unblinded) pooled variance estimate of the second stage (for $n_{2}=1$, we define $S_{2}^{2}=0$), $\Omega_{+}=\Omega_{a}+\Omega_{b}=2({\bar{X}}_{\cdot 1\cdot }-{\bar{X}}_{\cdot 2\cdot })$ be twice of the overall difference between the stages and $\Omega_{-}=\Omega_{a}-\Omega_{b}={\bar{\Delta }}_{1}-{\bar{\Delta }}_{2}$. With this notation, we can split the total variance estimate *S*^2^ into parts

(11) $$S^{2}=\frac{2n_{1}-2}{2n-2}S_{1}^{2}+\frac{2n_{2}-2}{2n-2}S_{2}^{2}+\frac{n_{1}n_{2}}{2n(2n-2)}\Omega_{+}^{2}+\frac{n_{1}n_{2}}{2n(2n-2)}\Omega_{-}^{2}$$

Obviously, $E(S_{2}^{2}|n)=E(S_{2}^{2})=\sigma^{2}$ for all *n*. As ${\bar{X}}_{\cdot 1\cdot },S_{1}^{2}$ and ${\bar{\Delta }}_{1}$ are mutually independent (and consequently *n* and ${\bar{X}}_{\cdot 1\cdot }$), and as ${\bar{X}}_{\cdot 1\cdot }$ and ${\bar{X}}_{\cdot 2\cdot }$ are independent under the condition of *n*, $\Omega_{+}$ is conditionally on *n* distributed as $N(0,2\sigma^{2}(1/n_{1}+1/n_{2}))$.

Under the condition of *n*, ${\bar{\Delta }}_{2}$ is distributed as $N(\delta ,2\sigma^{2}/n_{2})$. Further, under the condition of $S_{1}^{2}$ and ${\bar{\Delta }}_{1},\frac{n_{2}}{2\sigma^{2}}({\bar{\Delta }}_{1}-{\bar{\Delta }}_{2}{)}^{2}=\frac{n_{2}}{2\sigma^{2}}\Omega_{-}^{2}$ has a non-central $\chi^{2}$ distribution with 1 degree of freedom and non-centrality parameter $\frac{n_{2}({\bar{\Delta }}_{1}-\delta {)}^{2}}{2\sigma^{2}}$. Therefore, it follows

(12) $$\begin{array}{c} E(S^{2}-\sigma^{2}|S_{1}^{2},{\bar{\Delta }}_{1})=E[\frac{2n_{1}-2}{2n-2}(S_{1}^{2}-\sigma^{2})+\frac{2n_{2}-2}{2n-2}(S_{2}^{2}-\sigma^{2})\\ +\frac{\sigma^{2}}{2n-2}(\frac{n_{1}n_{2}}{2n\sigma^{2}}\Omega_{+}^{2}-1)+\frac{\sigma^{2}}{2n-2}(\frac{n_{1}}{n}\frac{n_{2}}{2\sigma^{2}}\Omega_{-}^{2}-1)|S_{1}^{2},{\bar{\Delta }}_{1}]\\ =E[\frac{n_{1}-1}{n-1}(S_{1}^{2}-\sigma^{2})+\frac{\sigma^{2}}{2n-2}(\frac{n_{1}}{n}\frac{n_{2}}{2\sigma^{2}}\Omega_{-}^{2}-1)|S_{1}^{2},{\bar{\Delta }}_{1}]\\ =E[\frac{n_{1}-1}{n-1}(S_{1}^{2}-\sigma^{2})+\frac{\sigma^{2}}{2n-2}(\frac{n_{1}}{n}+\frac{n_{1}n_{2}}{2\sigma^{2}n}({\bar{\Delta }}_{1}-\delta {)}^{2}-1)|S_{1}^{2},{\bar{\Delta }}_{1}]\\ =E[\frac{1}{n-1}((n_{1}-1)(S_{1}^{2}-\sigma^{2})+\frac{n_{2}}{2n}(\frac{n_{1}}{2}({\bar{\Delta }}_{1}-\delta {)}^{2}-\sigma^{2}))|S_{1}^{2},{\bar{\Delta }}_{1}] \end{array}$$

For cases $S_{1}^{2}$ and ${\bar{\Delta }}_{1}$ leading to $n_{2}=0$, we can go directly from the first to the last row in the above derivation.

Using $n=(1+r(S_{1}^{2},{\bar{\Delta }}_{1}))n_{1}$ and $n_{2}/n=(1+r(S_{1}^{2},{\bar{\Delta }}_{1}){)}^{-1}$, the bias $E(S^{2})-\sigma^{2}$ can therefore be written as

$$\begin{array}{c} \int_{0}^{\infty }\int_{-\infty }^{\infty }\frac{1}{\left(n_{1}-1+n_{1}r(q,t+\delta )\right)}((n_{1}-1)(q-\sigma^{2})+\frac{t^{2}n_{1}/2-\sigma^{2}}{2+2r(q,t+\delta )})\\ \times \phi_{0,\sqrt{2\sigma^{2}/n_{1}}}(t)\chi_{2n_{1}-2}^{2}(q\gamma )\gamma \text{d}t\text{d}q \end{array}$$

- The integral $\int_{-\infty }^{\infty }\ldots \text{d}t$ can again as in Theorem 1 be divided into $\int_{-\infty }^{0}\ldots \text{d}t$ and $\int_{0}^{\infty }\ldots \text{d}t$. By replacing *t* with −*t* in the first integral, it can be written as one integral over [0,∞] in the same way as in Theorem 1.
- One can conclude that the bias is symmetric around 0 for *δ* since r(q,y)=r(q,-y).
- The asymptotic unbiasedness for δ→±∞ follows from the integral expression with the dominated convergence theorem. □

### Proof of Theorem 4

For the proof of Theorem 4, we show the following lemmas: Lemma 1 Under the null hypothesis *δ* = 0

$$E(S_{1}^{2}|S_{1,\mathrm{OS}}^{2})=E[\frac{n_{1}}{2}{\bar{\Delta }}_{1}^{2}|S_{1,\mathrm{OS}}^{2}]=S_{1,\mathrm{OS}}^{2}$$

Proof $\frac{2n_{1}-1}{\sigma^{2}}S_{1,\mathrm{OS}}^{2}$ is $\chi^{2}$-distributed with $2n_{1}-1$ degrees of freedom and by equation (6) can be written as sum of $2n_{1}-1$ independent $\chi_{1}^{2}$-distributed random variables $Q_{j},j=1,\ldots ,2n_{1}-1,$ such that $Q_{1}=\frac{n_{1}}{2\sigma^{2}}{\bar{\Delta }}_{1}^{2}$ and $\sum_{j=2}^{2n_{1}-1}Q_{j}=\frac{2n_{1}-2}{\sigma^{2}}S_{1}^{2}$. All $Q_{1},\ldots ,Q_{2n_{1}-1}$ are identically distributed, and all *Q_j_* appear in the same way in the condition $S_{1,\mathrm{OS}}^{2}=\mathrm{const}\cdot \sum_{j=1}^{2n_{1}-1}Q_{j}$. Therefore, the *Q_j_* is exchangeable in the conditional distribution $E(Q_{1}|S_{1,\mathrm{OS}}^{2})$ without changing the value of it: we have $E(Q_{1}|S_{1,\mathrm{OS}}^{2})=E(Q_{j}|S_{1,\mathrm{OS}}^{2})$ for $j=2,\ldots ,2n_{1}-1$ and

$$\begin{array}{c} E(S_{1}^{2}|S_{1,\mathrm{OS}}^{2})=\frac{\sigma^{2}}{2n_{1}-2}\sum_{j=2}^{2n_{1}-1}E(Q_{j}|S_{1,\mathrm{OS}}^{2})=\sigma^{2}E(Q_{1}|S_{1,\mathrm{OS}}^{2})\\ =\frac{\sigma^{2}}{2n_{1}-1}\sum_{j=1}^{2n_{1}-1}E(Q_{j}|S_{1,\mathrm{OS}}^{2})=S_{1,\mathrm{OS}}^{2} \end{array}$$

□ Lemma 2 Let *c*, *d* be arbitrary with 0<c<d≤∞. For $S_{1,\mathrm{OS}}^{2}$, the following inequality is fulfilled

$$\text{Cov}\{S_{1,\mathrm{OS}}^{2},\frac{1}{\min(\max(S_{1,\mathrm{OS}}^{2},c),d)}\}>\text{Cov}(S_{1,\mathrm{OS}}^{2},\frac{1}{S_{1,\mathrm{OS}}^{2}})$$

Proof Note that a proof for d=∞ and for $S_{1}^{2}$ instead of $S_{1,\mathrm{OS}}^{2}$ is in Miller.^8^ To include the possibility of d<∞, we show

$$\begin{array}{c} \text{Cov}\{S_{1,\mathrm{OS}}^{2},\frac{1}{\min(\max(S_{1,\mathrm{OS}}^{2},c),d)}\}\\ =\text{Cov}\{S_{1,\mathrm{OS}}^{2},\max(\min(1/S_{1,\mathrm{OS}}^{2},1/c),1/d)\}\\ =\text{Cov}(S_{1,\mathrm{OS}}^{2},\frac{1}{S_{1,\mathrm{OS}}^{2}})-\text{Cov}\{S_{1,\mathrm{OS}}^{2},(1/S_{1,\mathrm{OS}}^{2}-1/c)\cdot 1_{\left\{S_{1,\mathrm{OS}}^{2}<c\right\}}\}\\ -\text{Cov}\{S_{1,\mathrm{OS}}^{2},(1/S_{1,\mathrm{OS}}^{2}-1/d)\cdot 1_{\left\{S_{1,\mathrm{OS}}^{2}>d\right\}}\} \end{array}$$

As $(1/x-1/c)\cdot 1_{\left\{x<c\right\}}$ and $(1/x-1/d)\cdot 1_{\left\{x>d\right\}}$ are monotonically decreasing functions of *x*, the second and third covariances above are ≤0; moreover, as *c* > 0, the second covariance is negative.^42^ This completes the proof. □ Proof of Theorem 4 From equation (12) in the proof of Theorem 3 follows with Lemma 1 under the null hypothesis *δ* = 0

$$\begin{array}{c} E(S^{2}-\sigma^{2})=E[E(S^{2}-\sigma^{2}|S_{1,\mathrm{OS}}^{2})]\\ =E[\frac{n_{1}-\frac{1}{2}-n_{1}/(2n)}{n-1}(S_{1,\mathrm{OS}}^{2}-\sigma^{2})] \end{array}$$

Since $E(S_{1,\mathrm{OS}}^{2})=\sigma^{2}$ and $\frac{n_{1}-1/2-n_{1}/(2n)}{n-1}$ as function of $S_{1,\mathrm{OS}}^{2}$ is a monotonically decreasing weight function, the bias of *S*^2^ is non-positive. Since there is no constant *c* such that $P(\frac{n_{1}-1/2-n_{1}/(2n)}{n-1}=c)=1$, the bias is negative and the upper bound is shown. To show the lower bound, we calculate (writing $n_{\min}=n_{1}+n_{2\min}$ and $n_{\max}=n_{1}+n_{2\max}$)

(13) $$E(S^{2}-\sigma^{2})$$

(14) $$=E[\frac{n_{1}-1/2}{n-1}(S_{1,\mathrm{OS}}^{2}-\sigma^{2})]-E[\frac{n_{1}}{2n(n-1)}(S_{1,\mathrm{OS}}^{2}-\sigma^{2})]$$

(15) $$>\frac{n_{1}-1/2}{v}E[\frac{S_{1,\mathrm{OS}}^{2}-\sigma^{2}}{\min\{(n_{\max}-1)/v,\max\{(n_{\min}-1)/v,S_{1,\mathrm{OS}}^{2}\}\}}]$$

(16) $$>\frac{n_{1}-1/2}{v}E(\frac{S_{1,\mathrm{OS}}^{2}-\sigma^{2}}{S_{1,\mathrm{OS}}^{2}})$$

(17) $$=\frac{n_{1}-1/2}{v}\{1-E(\frac{\sigma^{2}}{S_{1,\mathrm{OS}}^{2}})\}=-\frac{2n_{1}-1}{2n_{1}-3}\cdot \frac{1}{v}$$

The last expected value in equation (14) is negative since 1/(n(n-1)) as function of $S_{1,\mathrm{OS}}^{2}$ is monotonically decreasing; therefore, the total expression becomes smaller when this term is removed in equation (15). Further, we used in equation (15) the sample size formula $n_{2}=\min\{n_{2\max},\max\{n_{2\min},{\mathrm{vS}}_{1,\mathrm{OS}}^{2}-n_{1}+1\}\}$. For the inequality in equation (16), we applied Lemma 2. For the equality in equation (17), we have used $E(\sigma^{2}/S_{1,\mathrm{OS}}^{2})=E\{(2n_{1}-1)/X\}=(2n_{1}-1)E(X^{-1})=(2n_{1}-1)/(2n_{1}-3)$ where *X* is a chi-squared distributed random variable with $2n_{1}-1$ degrees of freedom. The expected value of $X^{-1}$ can be derived, for example, from Johnson et al.,^43^ p. 421.

When $\sigma^{2}\to \infty$, the last expected value in equation (14) converges to 0 which implies that the difference between the expressions in equations (14) and (15) converges to 0. Further, as the probability for $S_{1,\mathrm{OS}}^{2}>(n_{1}+n_{2\min})/v$ converges to 1 in the case $n_{2\max}=\infty$, also the difference between equations (15) and (16) converges to 0. Therefore, the lower bound becomes sharp when $\sigma^{2}\to \infty$. □

We note that in case of using the adjusted sample size rule $n_{2}^{a}$ instead of $n_{2}^{u}$, then we cannot go in a similar way from equations (15) to (16). Our simulation results in Figure 2 and in the lower panel of Figure 5 show that the bias for the adjusted sample size rule can be below the lower bound of Theorem 4.
