# Supplementary material for: Estimation after blinded sample size reassessment
Source: Stat Methods Med Res. 2016 Oct 2;27(6):1830–46. doi: 10.1177/0962280216670424 (PMC6047306; doi:10.1177/0962280216670424)
Supplement: Supplementary material [file Supplemental_material.pdf]

Supplementary Material

Estimation after blinded sample size reassessment

by Martin Posch, Florian Klinglmueller, Franz Koenig, and Frank Miller

In this document we present the numerical results shown in the article "Estimation after blinded sample size reassessment" as well as additional results that - for the sake of brevity - have not been included in the main article. Written in Rmarkdown this document serves both as supplementary material to the article but also as a program that permits a complete reproduction of all numerical results and graphical presentations of the results. This document is distributed as a vignette to the r-package *blindConfidence*. Therefore, all results shown in the paper as well as in this document can be replicated by compiling the corresponding source file in the package. Simply compiling the document - as happens on installation of the package - will recompute all figures using stored simulation results. To rerun the simulations the corresponding R chunks can be reconfigured by setting `eval=TRUE` so that they will be evaluated on compilation. Note that this may take a very long time and require a considerable amount of main memory. Computation of the publication results took over two weeks on a simulation server with 40 processor cores and 64 GB of shared memory. Note that the code has been optimized to run on Linux, so while the package should work fine on Windows in theory, it has not been tested.

In Section 1 we outline the setup and reproduce the results of the simulation study presented in Section 4 of the article. Additionally in Section 2 of this document show simulation results concerning the inflation of the non-coverage for the adjusted sample size rule, and the (squared) standard error of the mean estimate. In Section 3 we investigate the maximum absolute bias of the variance and mean estimates for given first stage sample sizes, as well as the maximum of the Type I error of the *t*-test following blinded sample size reassesement. In Section 4 we reproduce the simulations shown in the case study of our paper. In Section 5 we present a simulation study investigating the bias and coverage probabilities of estimaten and confidence intervals following blinded sample size reassessment when the second stage sample size is restricted to not exceed twice the preplanned second stage sample size (which in our example is equal to the first-stage sample size).

In this document we present 22 Figures (15 of which are not shown in the Article) which take up considerable space. To keep the text readable, we have pushed all figures to the end of document. The following table gives an index of which Figures in the original paper correspond to which in this document. Note that Figure 4 in the article combines three figures from this document into one panel.

Article.Figure	Supplementary.Figure
Figure 1	Figure 3
Figure 2	Figure 4
Figure 3	Figure 2
Figure 4	Figure 7, 13, 14
Figure 5	Figure 16

1. Coverage probabilities and bias of the mean and variance estimates

In a first simulation study we investigated the coverage probabilities of conventional t-test confidence intervals following blinded interim analysis, as well as the bias of the estimates of the mean and variance. The second stage sample size was computed based on the blinded first stage data using either the adjusted

$$n_2^a(S_{1,OS}^2) = \min\left\{n_{2\max}, \max\left\{n_{2\min}, 2(z_{1-\alpha}+z_{1-\beta})^2\left(\frac{S_{1,OS}^2}{\delta_0^2} - \frac{n_1}{4n_1-2}\right) - n_1 + 1\right\}\right\}.$$

or the unadjusted

$$n_2^u(S_{1,OS}^2) = \min\left\{n_{2\max}, \max\left\{n_{2\min}, 2(z_{1-\alpha}+z_{1-\beta})^2\frac{S_{1,OS}^2}{\delta_0^2} - n_1 + 1\right\}\right\},$$

reassessment rules.

To optimize the computational efficiency of the simulation algorithm, the joint distribution of the stage-wise t-statistics was simulated by sampling the stage-wise means and sample variances from independent normal and chi-squared distributions (instead of sampling individual observations). After submission of our manuscript we became aware of a paper by Lu (2016) on sample size reassessment for non-inferiority trials, where the same approach is proposed.

We assume that the preplanned sample size of the trial was planned to reach a target power of 80% (using the normal approximation) assuming an effect size $\delta_0 = 1$, standard deviations σ_0 between 0.5 and 2 in steps of .5 and a one-sided significance level $\alpha = 0.025$. The first stage sample size n_1 was set to halve of the preplanned sample size. For this simulation the maximum and minimum second stage sample sizes where not restricted (i.e. $n_{2\max} = \infty$, $n_{2\min} = 0$)The simulations were performed in R with $5 * 10^7$ simulation runs per scenario. The data used in the final manuscript version, is distributed with this package and can be loaded with the command `data(gridsim)`.

Difference between actual and nominal coverage probabilities

In Figure 1 we plot how far the coverage probabilities of one and two-sided confidence intervals deviate from their nominal coverage if sample sizes are reassessed using the adjusted variance estimator. In Figure 2 we show the difference between actual and nominal coverage probabilities, for designs that use the unadjusted lumped variance at interim for sample size reassessment (i.e. n_2^u).

Bias of the effect size estimate

Next we look at estimates of the mean difference between treatment groups. In Figure 3 solid lines show the bias in the mean estimate for designs where sample sizes are adjusted based on the adjusted interim variance estimate (n_2^a), dashed lines for designs where sample sizes are adjusted based on the unadjusted interim variance estimate (n_2^u). The dotted lines show the bounds for the bias of the mean estimate that can be attained under any (blinded) samplesize reassessment rule.

Bias of the variance estimate

In Figure 4 we show the bias when estimating the variance at the end of the adapted trial. Solid lines show the bias of the variance estimate for designs where sample sizes are adjusted based on the adjusted interim variance estimate (n_2^a), dashed lines for designs where sample sizes are adjusted based on the unadjusted interim variance estimate (n_2^u). The dotted lines show the bias of the mean estimate using the samplesize reassessment that maximizes the negative or positive bias, respectively.

Estimates of the variance of the mean estimate (squared standard error)

To compare the actual variance of the mean to the estimated variance, we computed for each simulated trial the variance estimate $\hat{S}_e^2 = \frac{2S^2}{n_1+n_2}$ as well as the actual variance $\sigma_f^2 = \frac{2\sigma^2}{n_1+n_2}$ of a design with fixed sample sizes n_1, n_2 (thus, ignoring that n_2 is dependent on the first stage sample). Both quantities were then averaged over the Monte-Carlo samples. Finally, we compute the variance of the mean estimate across all Monte Carlo samples, which is an unbiased estimate of the true variance of the mean (σ_e^2). The results show that the true variance of the mean estimate of an adaptive design with blinded sample size reassessment is smaller than the average variance of a fixed sample design with the same sample sizes. The bias of the estimate of the variance of the mean estimate is close to zero around the null hypothesis. This holds both for designs using the adjusted and unadjusted interim variance estimates to reassess the sample size and gives some intuition why there is no relevant inflation of the coverage probability of confidence intervals under the null hypotheses, even though the variance estimate has a considerable negative bias. We show $\hat{S}_e^2$, σ_e^2 and σ_f^2 for trials using the adjusted (Figure 5) and the adjusted sample size reassessment rules (Figure 6).

2. Maximum bias of the mean estimate and worst case inflation of the non-coverage

We also maximize the different absolute bias of mean and variance estimates as well as the (absolute) difference between actual and nominal coverage probabilities over δ and σ, for fixed first stage sample sizes. As the different quantities can only be evaluated with some simulation error, we implemented a three-stage optimization procedure.

1. We evaluate bias, and coverage probabilities using an initial simulation, for all per group first stage sample sizes between 2 and 50 over grid of parameter values $\delta \in [0, 4]$ and $\sigma \in [.5, 4]$ in steps of .1.
2. For each first stage sample size and quantity (mean bias, variance bias, actual - nominal coverage probabily) we identify the maximizing parameter settings based on the results of the simulation study in step one. We add additional candidate scenarios in the vicinity of each of the identified parameter settings (in a resolution that is higher then the grid in step 1) and perform another set of simulations (using an increased number of simulation runs).
3. Again, for each first stage sample size and quantity (mean bias, variance bias, actual - nominal coverage probabily) we identify the maximizing parameter settings based on the results of the simulation study in step two. Finally we run a last set of simulations using the identified scenarios to avoid any selection bias.

In the Figures 7-15 we present the results from the final simulation run in Step 3. For the bias of the variance we performed some additional simulations as our results suggested that the optimizing scenarios are given for $\delta = 0$ and for the undadjusted sample size rule also $\sigma = \infty$.

Step 1: Simulation over a grid

We simulate adaptive trials for true differences of means between 0 and 4, true standard deviations between .5 and 4 (both in steps of .1) and per group first stage sample sizes between 2 and 50. For each scenario we performed 10^6 simulation runs. Steps 2 and 3 of the maximizing procedure were then performed for each quantity seperately.

Step 2 and 3: Maximum bias of the mean for given n_1

To improve our estimate of the maximum bias of the mean estimate we refine the grid around the optima found in the simulation performed in Step 1 runs by adding points every .01 in a cubic vicinity - with a width

of .1 - of the maximizing δ and σ and simulate again using $4 * 10^6$ runs. We select the parameter values leading to the maximal bias and resimulate using 10^7 runs to remove any selection bias. The results for the maximum bias of the mean estimate are shown in Figure 7

Step 2 and 3: Maximum bias of the variance estimate

To improve our estimate of the maximum bias of the variance estimate we refine the grid around the optima found in the simulation performed in Step 1 runs by adding points every .01 in a cubic vicinity - with a width of .1 - of the maximizing δ and σ and simulate again using $4 * 10^6$ runs. We select the parameter values leading to the maximal bias and resimulate using 10^7 runs to remove any selection bias. The results of this simulation can be seen in Figure 13.

It appears that the absolute bias of the variance estimate (using both sample size rules) is maximised for $\delta = 0$. Above figure shows that in the simulation study the maximum is consistenly attained for δ close to zero. Also for the unadjusted reassessment rule, values of σ that maximise the absolute variance bias are consistently found at the upper border of the simulation grid. It appears that the maximum is obtained for large values of σ. Consequently we additionally ran the following simulations:

1. Maximize the absolute variance bias using a fixed value of $\delta = 0$, and values of σ ranging from 0.5 to 4
2. Estimate the absolute variance using simulations setting $\delta = 0$ and $\sigma = 4$ (i.e. at the border of the chosen parameter grid)
3. Estimate the absolute variance using simulations setting $\delta = 0$ and $\sigma = 50$ (i.e. approaching infinity)

Comparison of simulated optima for the unadjusted reassessment rule

In Figure 9 we compare the maximum of the absolute variance bias among scenarios where $\delta = 0$ is fixed to the maximum absolute bias among scenarios where δ was allowed to range from 0 to 4. It seems that the difference in the bias curves is due only to Monte Carlo error. We therefore conclude that the maximum is attained at $\delta = 0$.

In Figure 10 we compare the maximum of the absolute variance bias over scenarios where σ may range betwenn .5 and 4, where $\sigma = 4$ is fixed, and where σ is set to 50. We observe that - especially for smaller sample sizes - the absolute bias for scenarios with $\sigma = 50$ exceeds the maximum found for scenarios with $\sigma \leq 4$. The difference is consistent across different first stage sample sizes. Also, fixing $\sigma = 4$ does not result in a decrease of the absolute bias compared to settings where σ may range between .5 and 4 - but rather in a small but consistent increase[1]. In consequence we conclude that the maximum absolute bias of the variance (using the unadjusted sample size reassessment rule) is increasing in σ. In the remainder we will use $\sigma = 4$, which even though not optimal (in terms of maximising the bias) compared to $\sigma = 50$ (or even larger values) reflects a value that may be of practical relevance.

Comparison of simulated optima for the adjusted reassessment rule

Similar to the unadjusted sample size reassessment rule, the results shown in Figure 11 suggest that the difference between the maximum absolute variance bias found for scenarios where $\delta = 0$ fixed, and scenarios where δ was allowed to range from 0 to 4 is due only to Monte Carlo error. We therefore conclude that the maximum is attained at $\delta = 0$.

In Figure 12, we show that, for the adjusted sample size rule, setting $\delta = 0$ and $\sigma = 50$ decreases the absolute variance bias compared to the maximum values found when $\delta = 0$ and σ ranged from .5 to 4; and also, that setting $\sigma = 4$ does decrease the bias compared to the maximum values found when $\delta = 0$ and σ ranged from .5 to 4, but not as much as for $\sigma = 50$. The differences are consistent across different first stage sample sizes and appear larger than the Monte Carlo Error. We therefore conclude that the the (absolute) bias is maximized for values of σ lying within the parameter grid.

[1]This may seem as surprising as the $\sigma = 4$ is included in the scenario where σ is not fixed. However, if the local optimum is attained at $\sigma = 4$ optimizing over a range of values is likely to select a different (suboptimal) value due to Monte Carlo error, thus resulting in a smaller estimate of the maximum (absolute) bias.

Final results included in the paper

Based on above results we concluded to present the results that show the maximum absolute variance bias for $\delta = 0$ and σ ranging from .5 to 4 - when using the adjusted sample size rule; and $\delta = 0$ and $\sigma = 4$ - when using the unadjusted sample size rule. Even though not a global maximum we opted for the latter scenario as it reflects the maximum absolute variance bias within a range of parameters that seem of practical concern. The corresponding results are shown in Figure 13.

Step 2 and 3: Maximum difference between nominal and actual coverage probability

To improve our estimate of the absolute difference between nominal and actual coverage probability we refine the grid around the optima found in the simulation performed in Step 1 runs by adding points every .005 in a cubic vicinity - with a width of .4 - of the maximizing δ and σ and simulate again using $4 * 10^6$ runs. We select the parameter values leading to the maximal bias and resimulate using 10^7 runs to remove any selection bias. The results of this simulation can be seen in Figure 14.

Maximimizing the inflation of the non-coverage probability under the Null

We also performed a simulation based optimization of the inflation of the non-coverage probability under the null hypotheses (i.e. $\delta = 0$). This corresponds to the inflation of the Type I error rate of the associated test procedure. The results show that there is minor inflation of the Type I error rate for first stage per group sample sizes below 10 (.5 to .01 percentage points). For larger sample sizes the inflation is less than the simulation error. See Figure 15 for the corresponding results.

3. Case Study

As an example Kieser and Friede (2003) present the trial reported in Malsch and Kieser (2001), which is a randomized placebo controlled trial of the efficacy of the kava-kava special extract "WS$^{\H{o}}$ 1490" for the treatment of anxiety. The primary endpoint of the study was the change in the Hamilton Anxiety Scale (HAMA) between baseline and end of treatment. Assuming a mean difference between treatment and control of $\delta_0 = 5.5$ and standard deviation of $\sigma_0 = 8$ (i.e. a variance of $\sigma_0^2 = 64$) results in a sample size of 34 patients per group to provide a power of 80%.

Kieser and Friede (2003) assume that sample size reassessment based on blinded data is performed after 15 patients per group and consider that the interim estimate of the standard deviation is $S_{1,OS} = 6$ and adjusting for $\delta_0 = 5.5$ gives $S_{1,OS,\delta_0} = 5.3$. The corresponding updated sample sizes are then given by $n_2^u = 4.7$ and $n_2^a = 0.6$ patients (per group), respectively. Consequently, using the unadjusted sample size rule 5 patients per group would have been recruited in the second stage; using the adjusted rule only 1 patient per group would have been recruited in the second stage.

If the sample size reassessment is based on the unadjusted interim variance estimate $S_{1,OS}$, the variance estimate from the completed trial will be negatively biased. Theorem 4 gives a lower bound for the magnitude of that bias which in this case is -2.07. We also performed a simulation study, similar to that of Section 4 in the paper, where we fix $\delta_0 = 5.5$, $\sigma_0 = 8$ and $n_1 = 15$ - but not $S_{1,OS}$, S_{1,OS,δ_0} - and let δ vary from $-2\delta_0 = -11$ to $2\delta_0 = 11$ in steps of 0.05 and σ from 1 to 20 in steps of 1. Detailed results are shown in Figure 16 where we plot the coverage of the confidence intervals and biases of the mean, and the variance estimate. The variance bias gets as low as -2.06 if the true mean effect is $\delta = -0.82$ and the true standard deviation $\sigma = 19$ (i.e. it reaches the theoretical boundary within simulation error). For smaller values of the true standard deviation the bias is smaller in magnitude. The mean bias goes up to 0.2 for negative values of the true effect size and as low as -0.2 for positive values of the true effect size. The absolute bias reaches its maximum for a true mean differences of $\delta = \pm 7.98$) and standard deviation of $\sigma = 5$). The maximum inflation of the coverage probabilities of one-sided 97.5% and two-sided 95% confidence intervals is 0.7 and

0.5 percentage points, respectively. The actual coverage probabilities are smallest for large absolute values of the true mean difference and standard deviations of around 5.

If the sample size reassessment is based on the adjusted variance estimate, the absolute bias of the variance and mean estimate will be even larger taking values up to 2.49 for the variance and up to 0.25 for the mean, respectively. The inflation of the coverage probabilities goes up to 0.9 percentage points for the one-sided confidence intervals and 0.6 percentage points for the two-sided intervals.

4. Restricted sample size rules

Additionally we investigate the effect of blinded sample size reassessment on the coverage probabilities of conventional t-test confidence intervals, as well as the bias of the estimates of the mean and variance, when the second stage sample size is limited by a predetermined upper bound. Specifically we compute the second stage sample size using the rules n_2^a and n_2^u as defined above, setting the maximum second stage sample size to $n_{2\max} = 2n_1$, but leaving the minimum sample size at zero (i.e. $n_{2\min} = 0$). Because the (blinded) interim analysis is performed after one half of the preplanned total (per-group) sample size is observed, setting $n_{2\max} = 2n_1$ is equivalent to limiting the second stage sample size to be at most twice its preplanned value.

As in Section 1 we assume that the preplanned sample size of the trial was planned to reach a target power of 80% (using the normal approximation) assuming an effect size $\delta_0 = 1$, standard deviations σ_0 between 0.5 and 2 in steps of .5 and a one-sided significance level $\alpha = 0.025$. The upper bound for the second stage per group sample size $n_{2\max}$ was set to twice the preplanned second stage sample size (i.e. $n_{2\max} = 2n_1$). The simulations were performed in R with 10^7 simulation runs per scenario. The data used in the final manuscript version, is distributed with this package and can be loaded with the command `data(restsim)`.

Overall we observe that for $\sigma_0 < \sigma$ the bias of effect and variance estimates (Figures 19 and 20, respectively) as well as the difference (in absolute terms) between nominal and actual coverage probabilities (Figures 17 and 18) is substantially reduced. This is not surprising as for $\sigma_0 << \sigma$ the adjusted second stage sample size is essentially fixed at $n_{2\max}$ resulting in unbiased estimates and correct coverage probabilities. Note, however, that the corresponding trials will not control the Type II error at the desired level anymore. This can also be seen as the variance of the mean estimate (Figures 21 and 22) is substantially larger in these cases and does not decrease for increasing absolute effect sizes, compared to the unrestricted samplesize reassessment rules.

For $\sigma_0 \geq \sigma$ we observe that the shape of the bias of estimates and the difference between nominal and actual coverage probabilities as functions of the true effect size is similar to when unrestricted sample size rules are applied. While the (absolute) bias of the effect estimate is reduced by about a factor $n_{2\max}/(n_1 + n_{2\max}) = 2/3$ (following from Theorem 2 in the article) the bias of the variance estimate is comparable in magnitude to when unrestricted sample size reassessment rules are applied. The effect on the difference between nominal and actual coverage probabilities is not as clear cut. For σ close to σ_0 there appears to be a reduction (to about 80%) compared to designs without sample size restrictions, for $\sigma < \sigma_0$ the magnitude of the differences is similar.

Figures

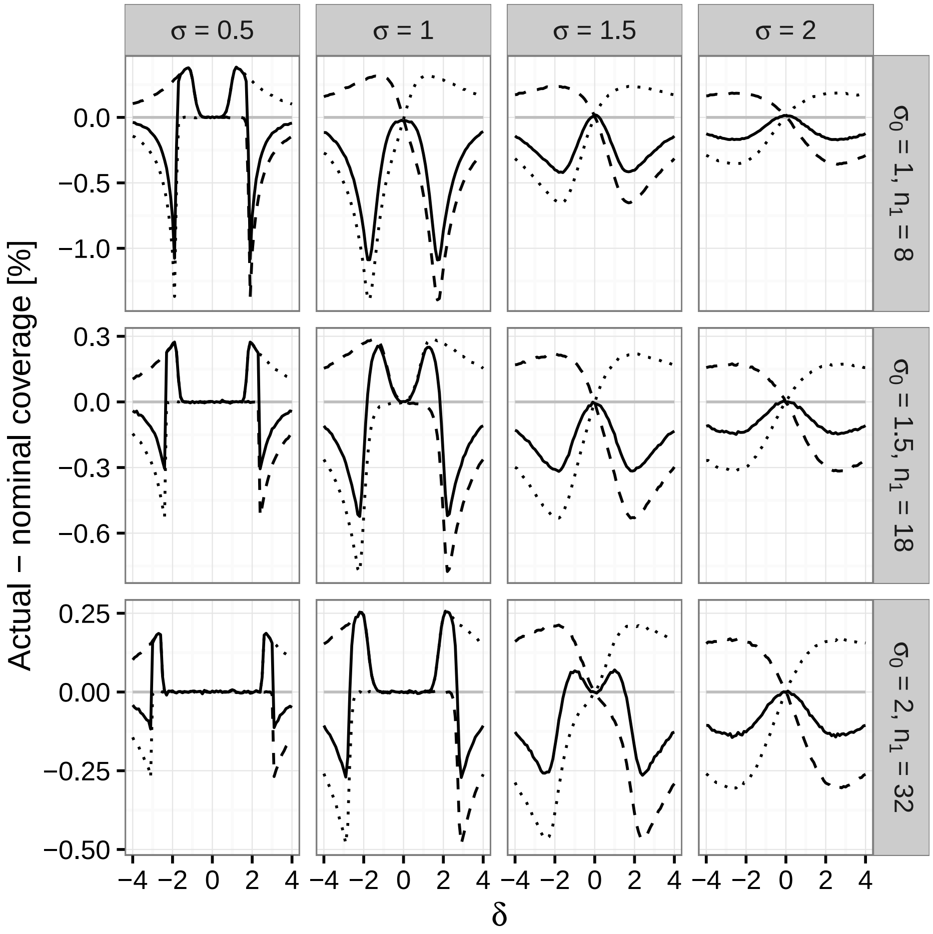

Figure 1: Difference between actual and nominal coverage probabilities in percentage points under blinded sample size reassessment using the *adjusted sample size reassessment rule* n_2^a: upper confidence bound (dashed line), lower confidence bound (dotted line) and two-sided interval (solid line). Rows refer to the a priori assumed standard deviations σ_0 determining the first stage sample size n_1. The columns correspond to actual standard deviations. The x-axis in each graph denotes the true treatment effect δ, the y-axis shows the difference of actual to nominal coverage probability such that negative values indicate settings where the confidence bound is not valid.

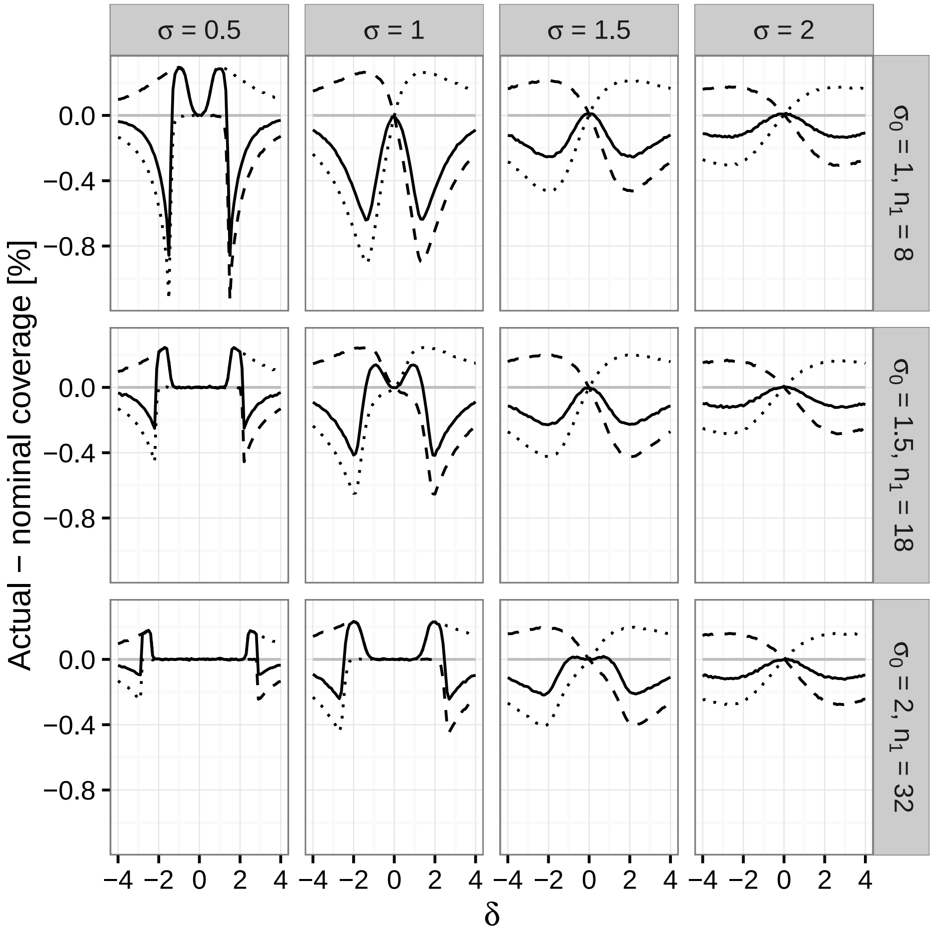

Figure 2: Difference between actual and nominal coverage probabilities in percentage points under blinded sample size reassessment using the *unadjusted interim variance estimate* $S_{1,OS}$: upper confidence bound (dashed line), lower confidence bound (dotted line) and two-sided interval (solid line). Rows refer to the a priori assumed standard deviations σ_0 determining the first stage sample size n_1. The columns correspond to actual standard deviations. The x-axis in each graph denotes the true treatment effect δ, the y-axis shows the difference of actual to nominal coverage probability such that negative values indicate settings where the confidence bound is not valid.

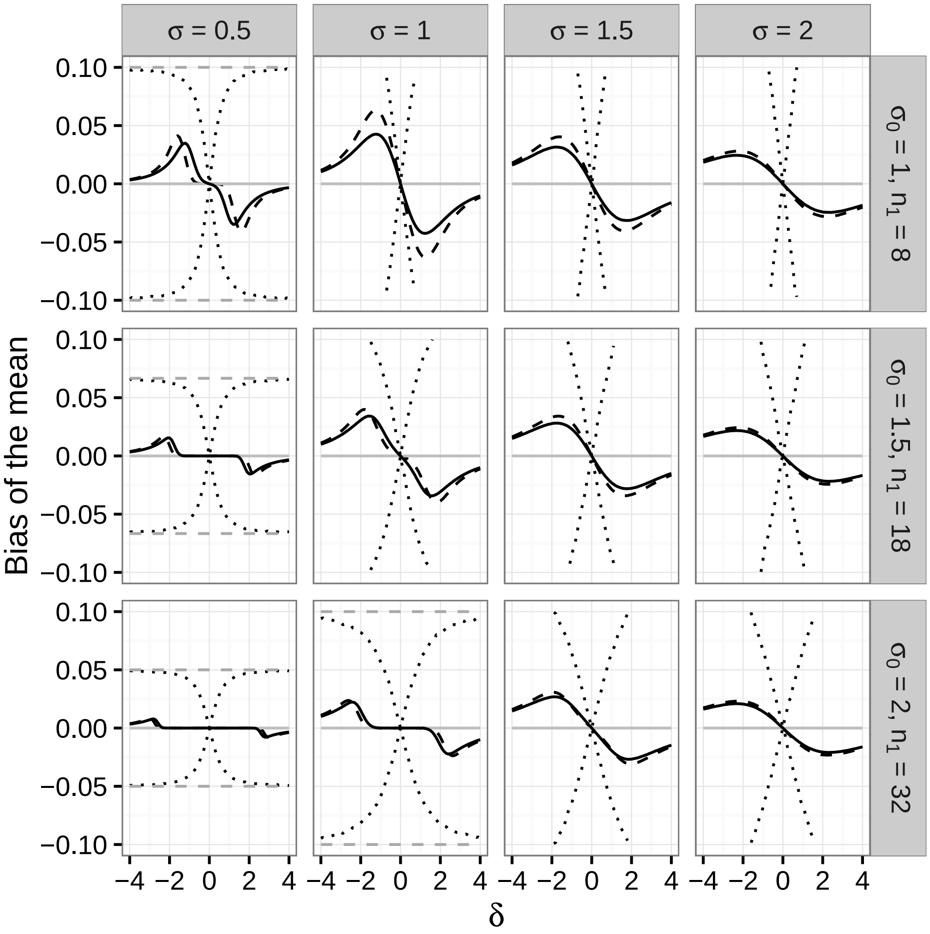

Figure 3: Bias of the mean under blinded sample size reassessment using the unadjusted (solid line) and adjusted (dashed line) interim variance estimate. The dotted lines show maximum negative and positive bias that can be attained under any blinded sample size reassessment rule according to Theorem 2. The dashed gray line shows the maximum bias that can be attained under any (unblinded) sample size reassessment rule. The treatment effect used for planning is set to $\delta_0 = 1$. Rows refer to the a priori assumed standard deviations σ_0 determining the first stage sample size n_1. The columns correspond to actual standard deviations. The x-axis in each graph denotes the true treatment effect δ, the y-axis shows the bias.

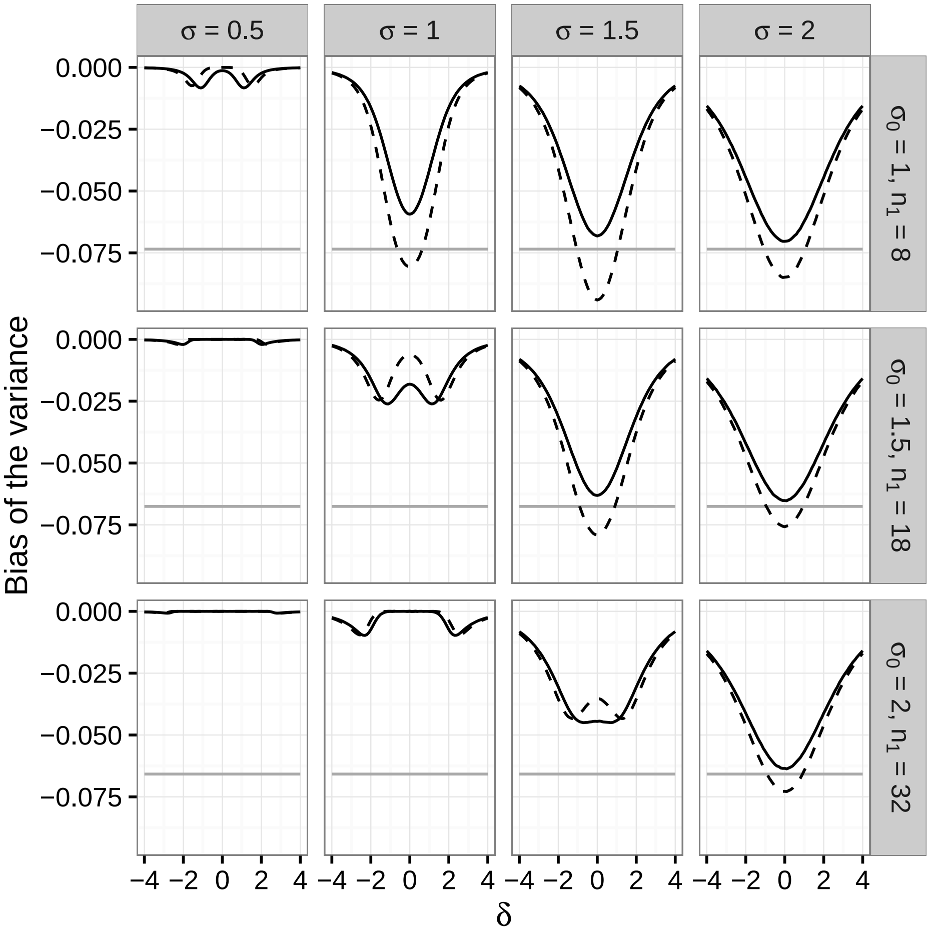

Figure 4: Bias of the variance under blinded sample size reassessment using the unadjusted (solid line) and adjusted (dashed line) interim variance estimate. The gray line gives the lower bound from Theorem 4 for the bias under sample size reassessment based on the unadjusted variance estimate. The treatment effect used for planning is set to $\delta_0 = 1$. Rows refer to the a priori assumed standard deviations σ_0 determining the first stage sample size n_1. The columns correspond to actual standard deviations. The x-axis in each graph denotes the true treatment effect δ, the y-axis shows the bias.

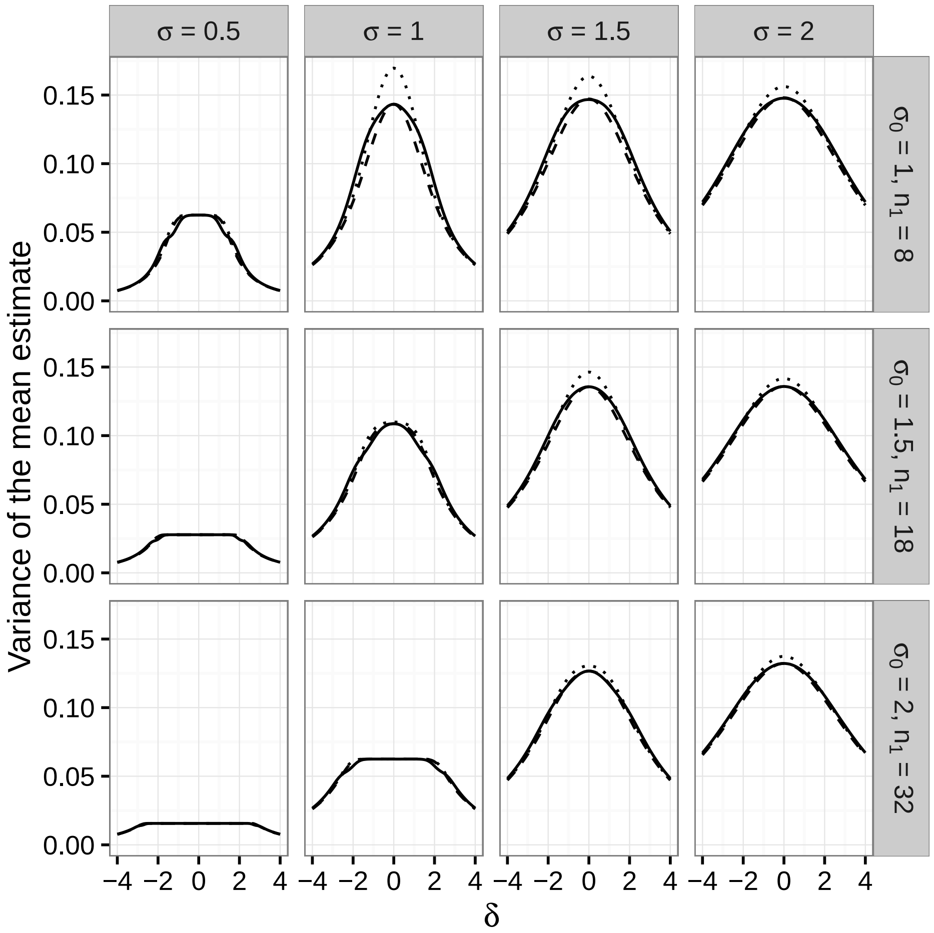

Figure 5: Adjusted sample size reassessment rule: actual variance of the mean estimate σ_e^2 (solid line), average of the estimated variances of the mean S_e^2 (dashed line), average of the variances σ_f^2 of the mean of fixed sample trials with the same sample sizes.

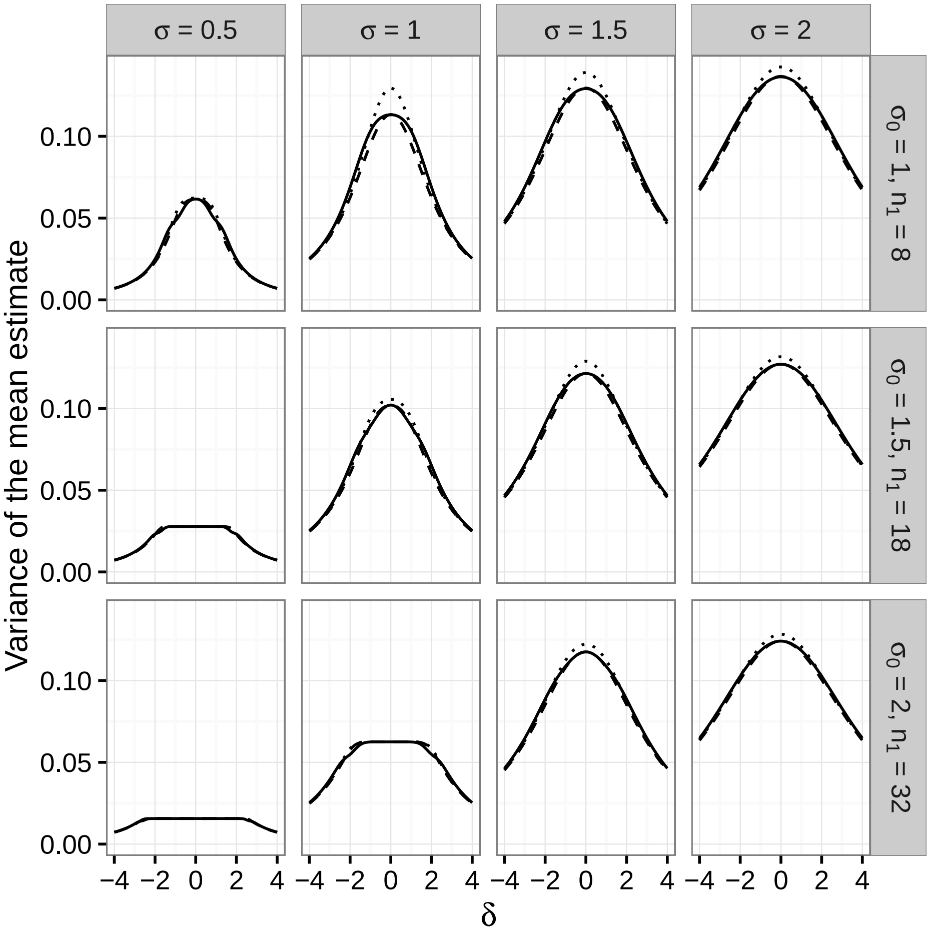

Figure 6: Variance of the effect estimates (i.e., square of the standard errors) for the unadjusted sample size reassessment rule: actual variance σ_e^2 of the mean estimate (solid line), average of the estimated variances S_e^2 of the mean (dashed line), average of the variance σ_f^2 of the mean of corresponding fixed sample trials (dotted line).

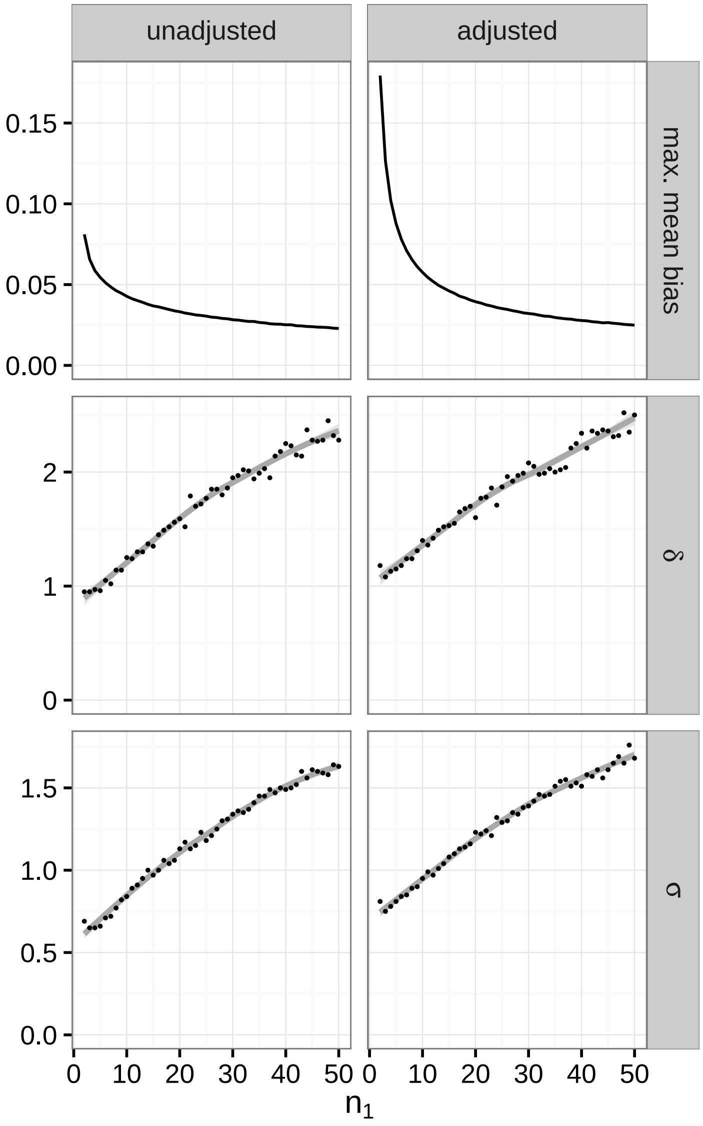

Figure 7: Maximum absolute bias of the mean estimate for given per group first stage sample sizes n_1 between 2 and 50. Left column shows the results for the unadjusted sample size reassessment rule, right column for the adjusted sample size reassessment rule. The first row shows the value of the bias, the second the effect size δ and the third the standard deviation σ at which the specific value is attained. The gray line denotes a Loess smoothed estimate.

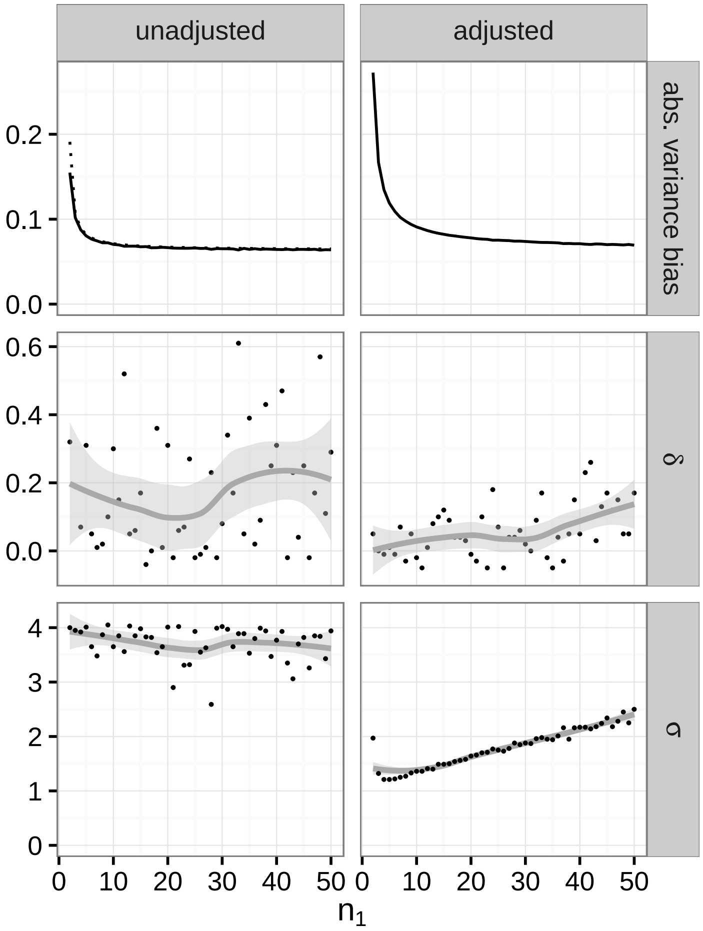

Figure 8: Maximum absolute bias of the variance estimate for given per group first stage sample sizes n_1 between 2 and 50. Left column shows the results for the unadjusted sample size reassessment rule, right column for the adjusted sample size reassessment rule. The first row shows the value of the bias, the second the effect size δ and the third the standard deviation σ at which the specific value is attained. The gray line denotes a Loess smoothed estimate.

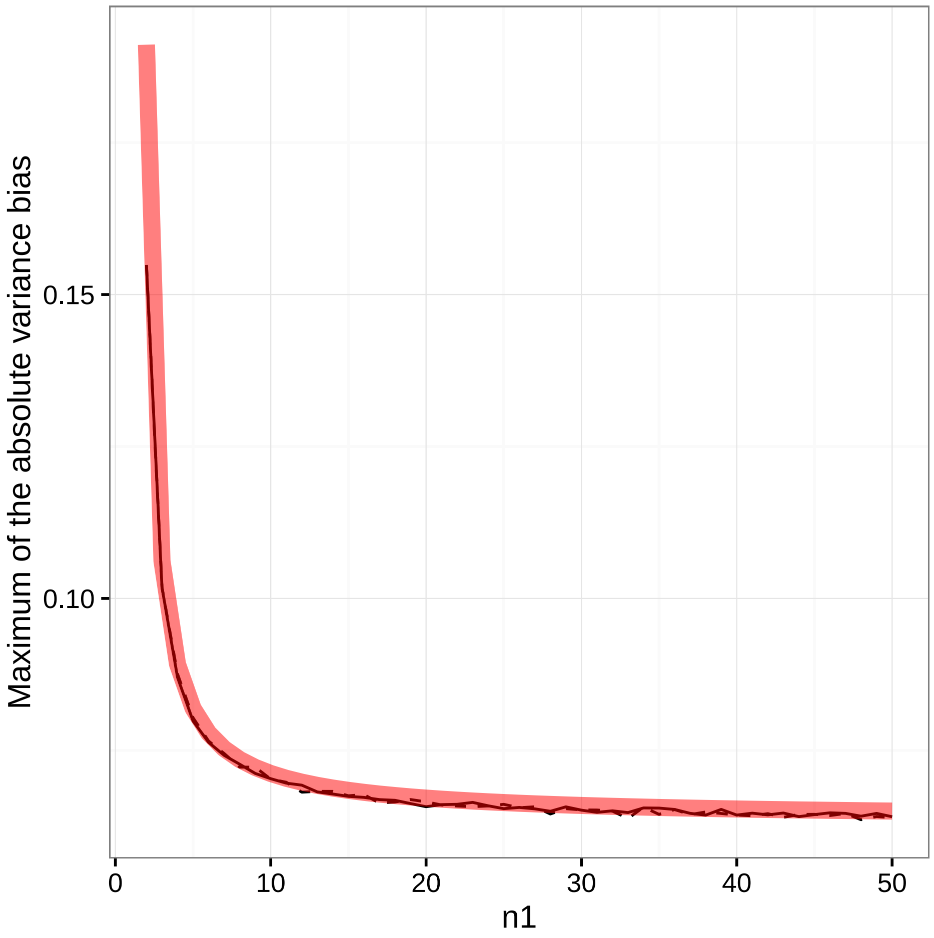

Figure 9: Comparison of maximal absolute variance bias for different parameter settings: Solid line - $\delta = 0$, $\sigma \in [.5, 4]$; Dashed line - $\delta \in [0, 4]$, $\sigma = [.5, 4]$; transparent red line - theoretical bound.

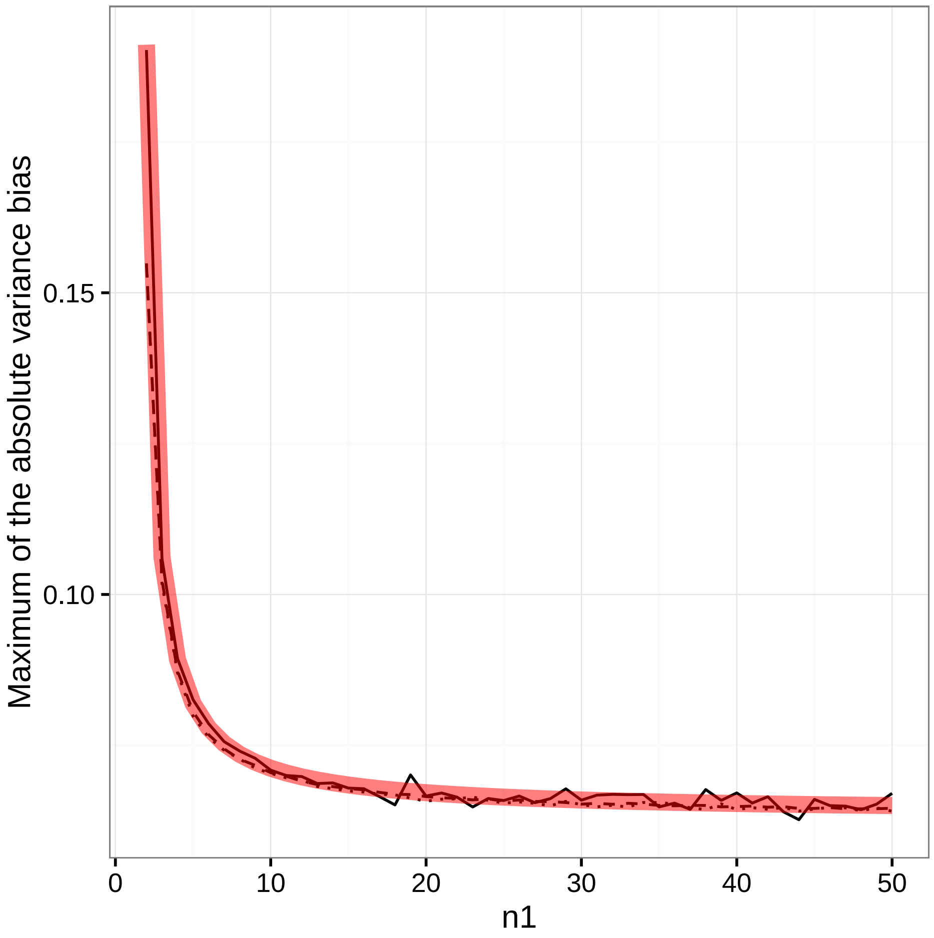

Figure 10: Comparison of maximal absolute variance bias for different parameter settings: Solid line - $\delta = 0$, $\sigma = 50$; Dashed line - $\delta = 0$, $\sigma = 4$; Dotted line $\delta = 0$, $\sigma \in [.5, 4]$; transparent red line - theoretical bound.

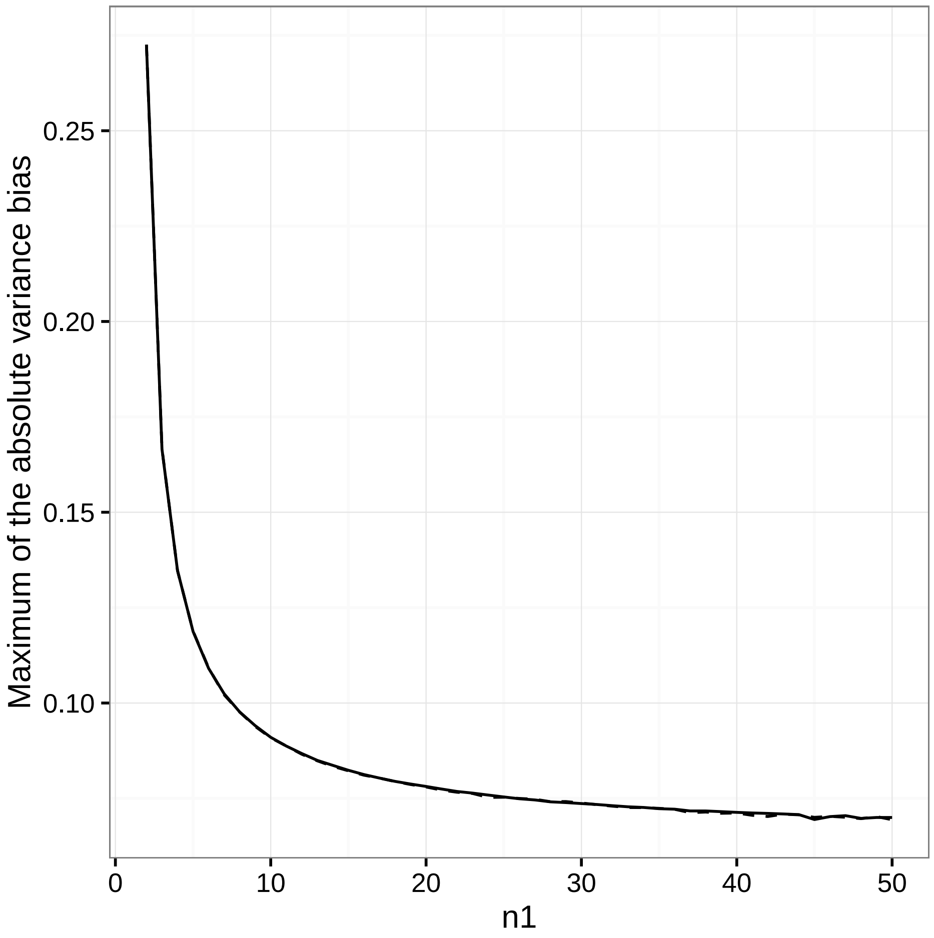

Figure 11: Comparison of maximal absolute variance bias for different parameter settings: Solid line - $\delta = 0$, $\sigma \in [.5, 4]$; Dashed line - $\delta \in [0, 4]$, $\sigma = [.5, 4]$.

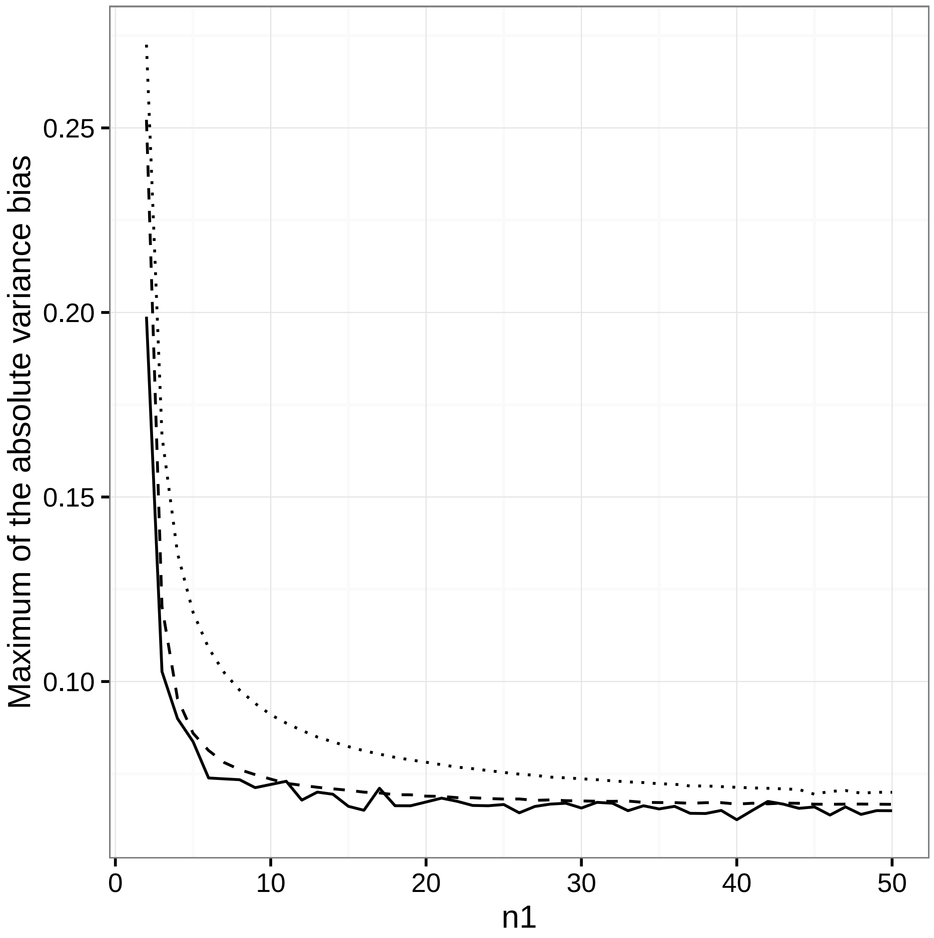

Figure 12: Comparison of maximal absolute variance bias for different parameter settings: Solid line - $\delta = 0$, $\sigma = 50$; Dashed line - $\delta = 0$, $\sigma = 4$; Dotted line $\delta = 0$, $\sigma \in [.5, 4]$.

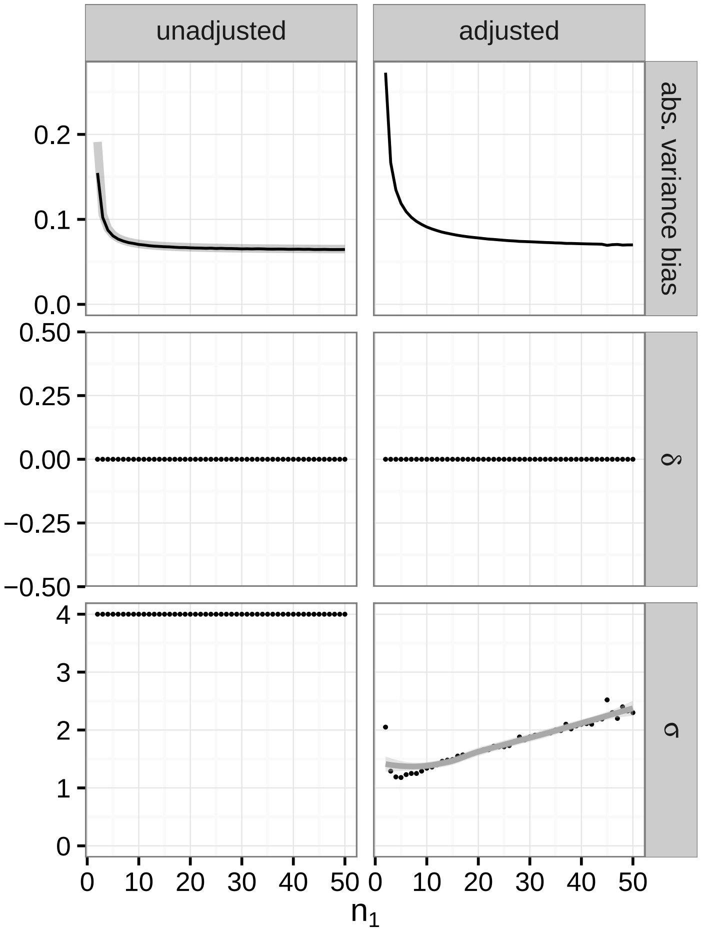

Figure 13: Maximum absolute bias of the variance estimate for given per group first stage sample sizes n_1 between 2 and 50 using fixed values $\delta = 0$ and for the unadjusted reassessment rule also $\sigma = 4$. Left column shows the results for the unadjusted sample size reassessment rule, right column for the adjusted sample size reassessment rule. The first row shows the value of the bias, the second the effect size δ and the third the standard deviation σ at which the specific value is attained. The gray line denotes a Loess smoothed estimate.

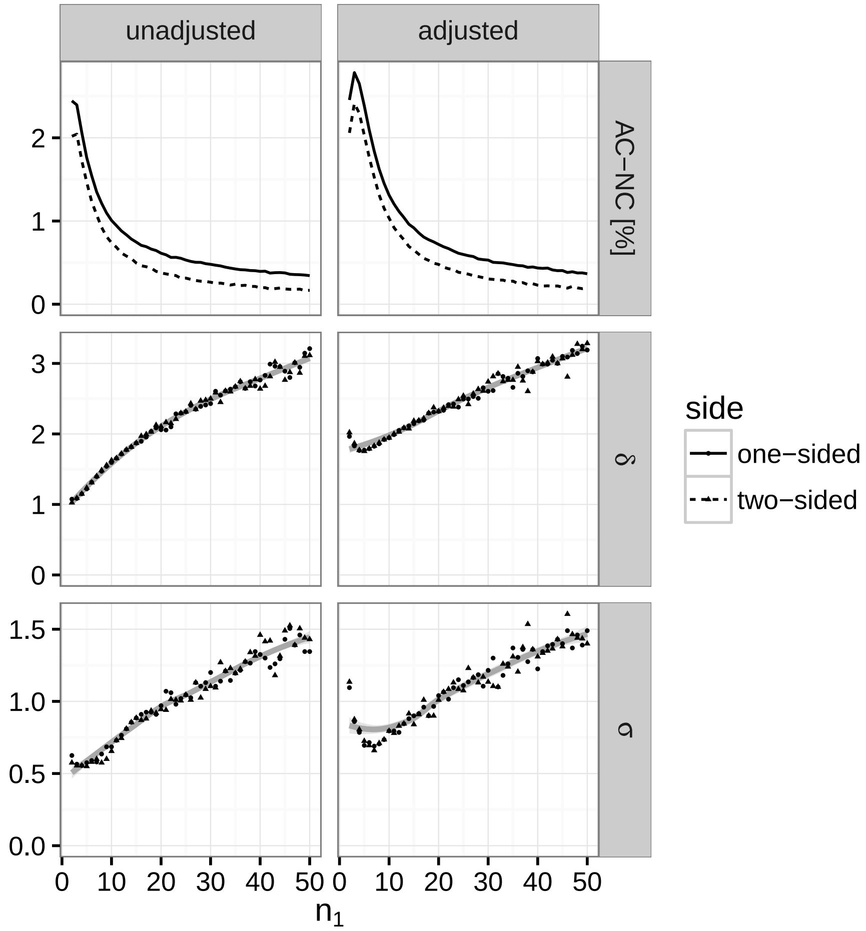

Figure 14: Maximum (absolute) difference between actual and nominal coverage probabilities for a given per group first stage sample sizes n_1 between 2 and 50. Left column shows the results for the unadjusted sample size reassessment rule, right column for the adjusted sample size reassessment rule. The first row shows the difference between actual and nominal coverage probabilities, the second the effect size δ and the third the standard deviation σ at which the specific value is attained. The gray line denotes a Loess smoothed estimate.

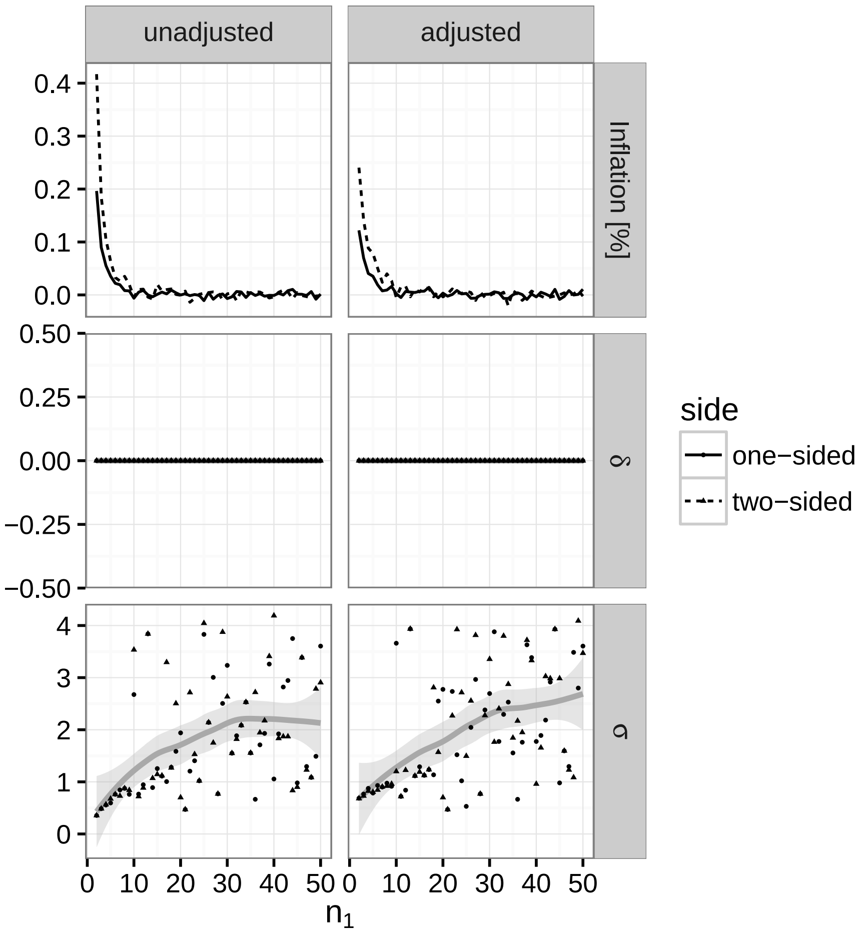

Figure 15: Maximum Type I error rate inflation of the t-test following blinded sample size reassessment based on blinded interim data for given per group first stage sample sizes n_1 between 2 and 50. Left column shows the results for the unadjusted sample size reassessment rule, right column for the adjusted sample size reassessment rule. The first row shows the difference between actual and nominal coverage probabilities, the second the effect size δ and the third the standard deviation σ at which the specific value is attained. The gray line denotes a Loess smoothed estimate.

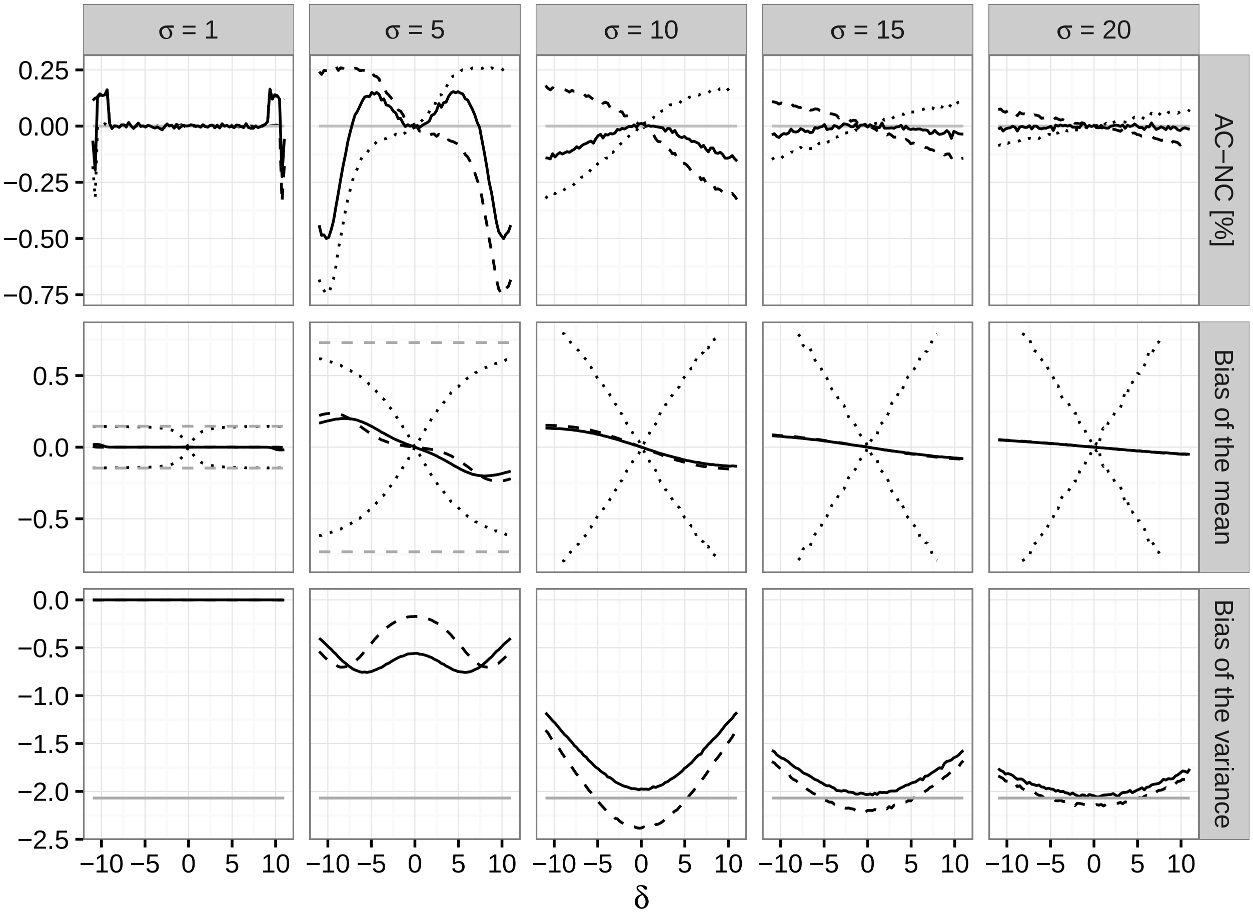

Figure 16: Coverage probabilities and bias of mean and variance estimates for the case study. The first panels shows actual - nominal coverage probabilities (AC-NC) for the 97.5% upper (dashed line), lower (dotted line) and the 95% two-sided confidence intervals, for the unadjusted reassessment rule n_2^u. The second panel shows the bias of the mean estimate if n_2^u (solid line) or n_2^a (dashed line) is used. The dotted line shows upper and lower bounds for the bias for general blinded sample size reassessment rules based on $S^2_{1,OS}$. The dashed gray line shows the bounds for the bias that can be attaind with a general unblinded sample size reassessment rule. The third panel shows the bias of the variance estimate if either n_2^u (solid line) or n_2^a (dashed line) is used. The red line shows the theoretical boundary for the bias give in Theorem 4.

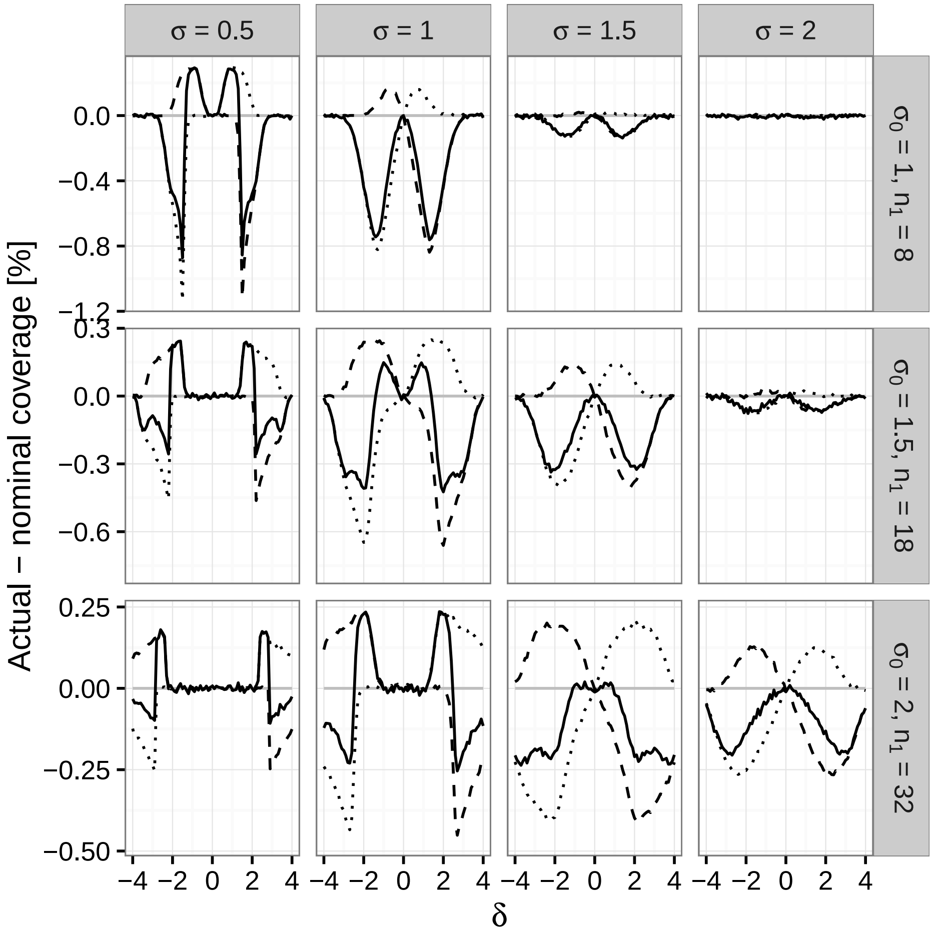

Figure 17: Difference between actual and nominal coverage probabilities in percentage points under blinded sample size reassessment using the *adjusted sample size reassessment rule* n_2^a with a maximal second stage sample size limited to $2n_1$: upper confidence bound (dashed line), lower confidence bound (dotted line) and two-sided interval (solid line). Rows refer to the a priori assumed standard deviations σ_0 determining the first stage sample size n_1. The columns correspond to actual standard deviations. The x-axis in each graph denotes the true treatment effect δ, the y-axis shows the difference of actual to nominal coverage probability such that negative values indicate settings where the confidence bound is not valid.

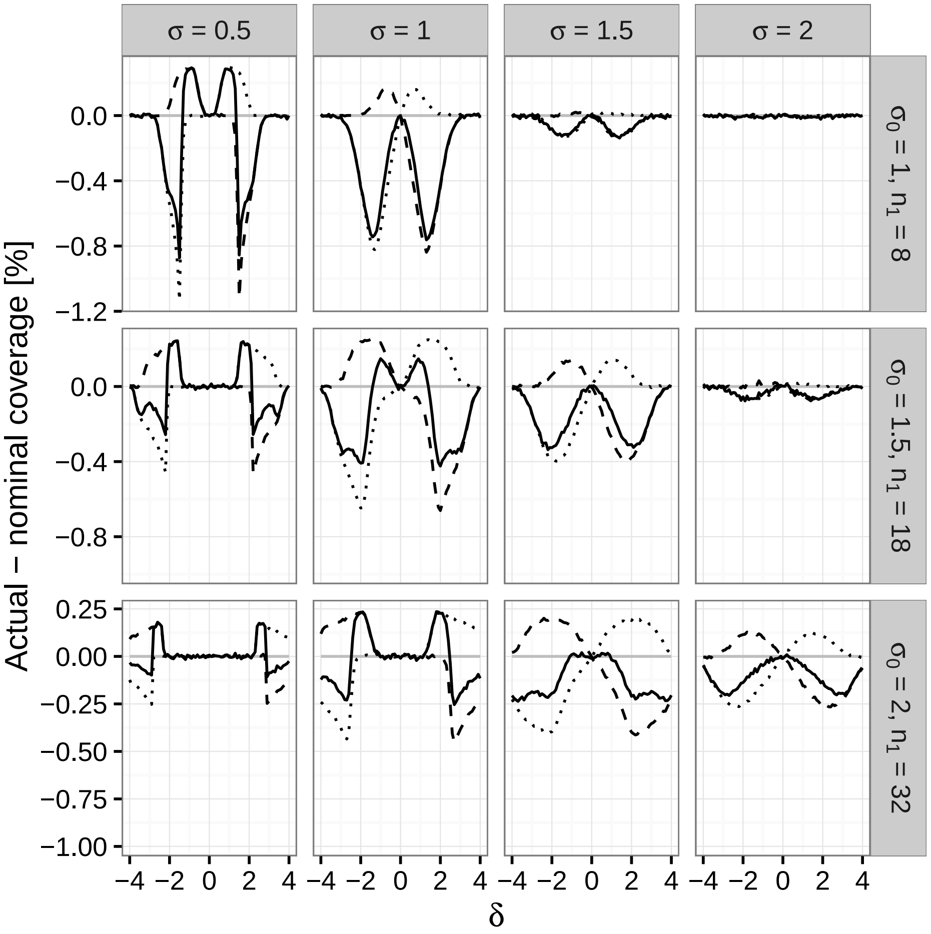

Figure 18: Difference between actual and nominal coverage probabilities in percentage points under blinded sample size reassessment using the *unadjusted interim variance estimate* $S_{1,OS}$ with a maximal second stage sample size limited to $2n_1$: upper confidence bound (dashed line), lower confidence bound (dotted line) and two-sided interval (solid line). Rows refer to the a priori assumed standard deviations σ_0 determining the first stage sample size n_1. The columns correspond to actual standard deviations. The x-axis in each graph denotes the true treatment effect δ, the y-axis shows the difference of actual to nominal coverage probability such that negative values indicate settings where the confidence bound is not valid.

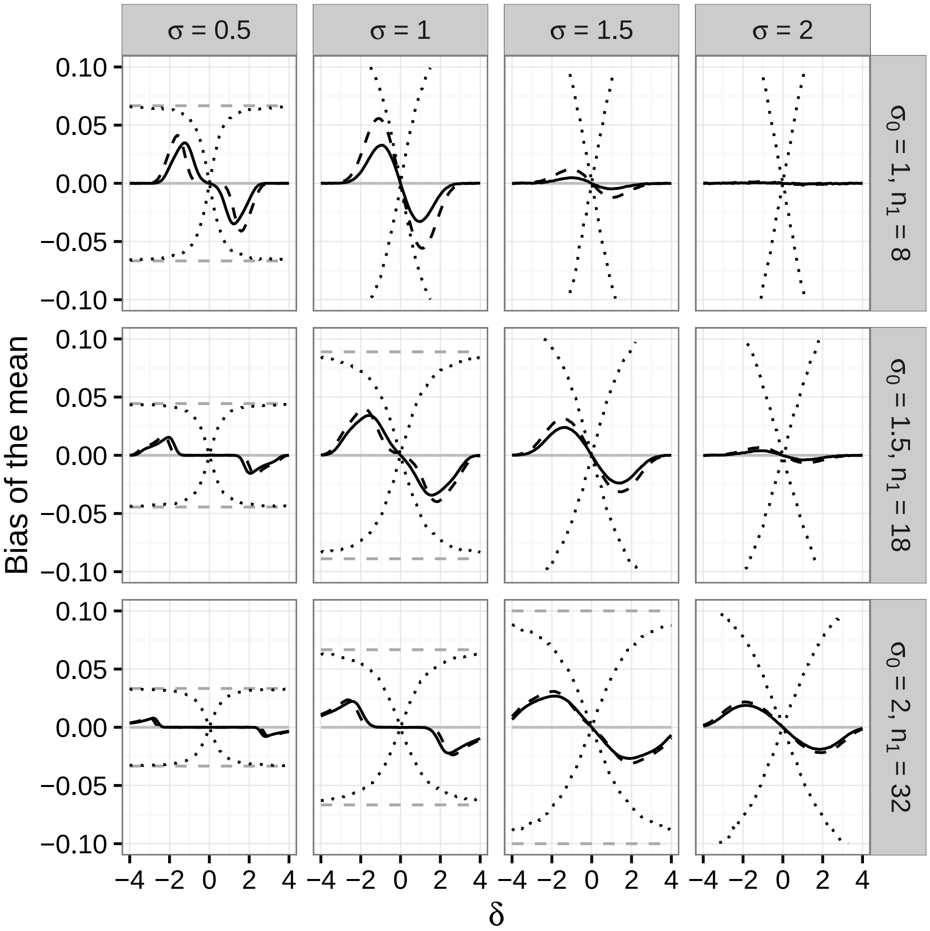

Figure 19: Bias of the mean under blinded sample size reassessment with a maximal second stage sample size limited to $2n_1$ using the unadjusted (solid line) and adjusted (dashed line) interim variance estimate. The dotted lines show maximum negative and positive bias that according to Theorem 2 can be attained under any blinded sample size reassessment rule with a maximal second stage sample size limited to $2n_1$. The dashed gray line shows the maximum bias that can be attained under any (unblinded) sample size reassessment rule with a maximal second stage sample size limited to $2n_1$. The treatment effect used for planning is set to $\delta_0 = 1$. Rows refer to the a priori assumed standard deviations σ_0 determining the first stage sample size n_1. The columns correspond to actual standard deviations. The x-axis in each graph denotes the true treatment effect δ, the y-axis shows the bias.

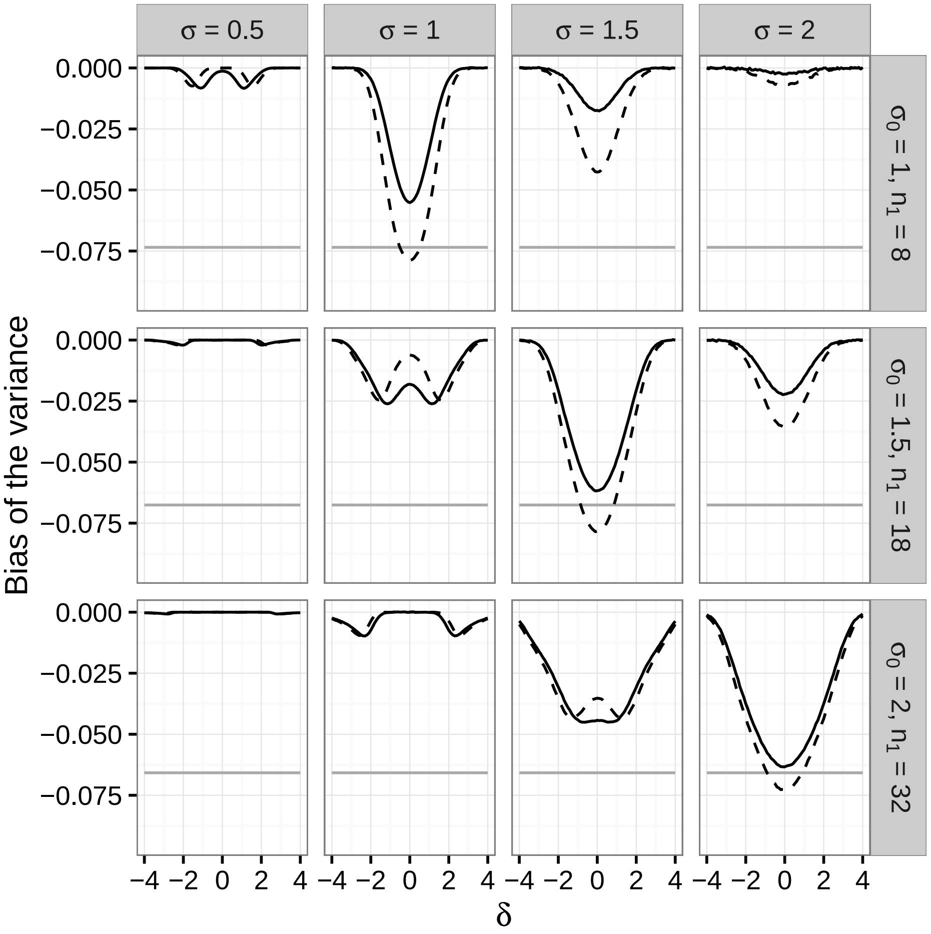

Figure 20: Bias of the variance under blinded sample size reassessment with a maximal second stage sample size limited to $2n_1$ using the unadjusted (solid line) and adjusted (dashed line) interim variance estimate. The gray line gives the lower bound from Theorem 4 for the bias under sample size reassessment based on the unadjusted variance estimate. The treatment effect used for planning is set to $\delta_0 = 1$. Rows refer to the a priori assumed standard deviations σ_0 determining the first stage sample size n_1. The columns correspond to actual standard deviations. The x-axis in each graph denotes the true treatment effect δ, the y-axis shows the bias.

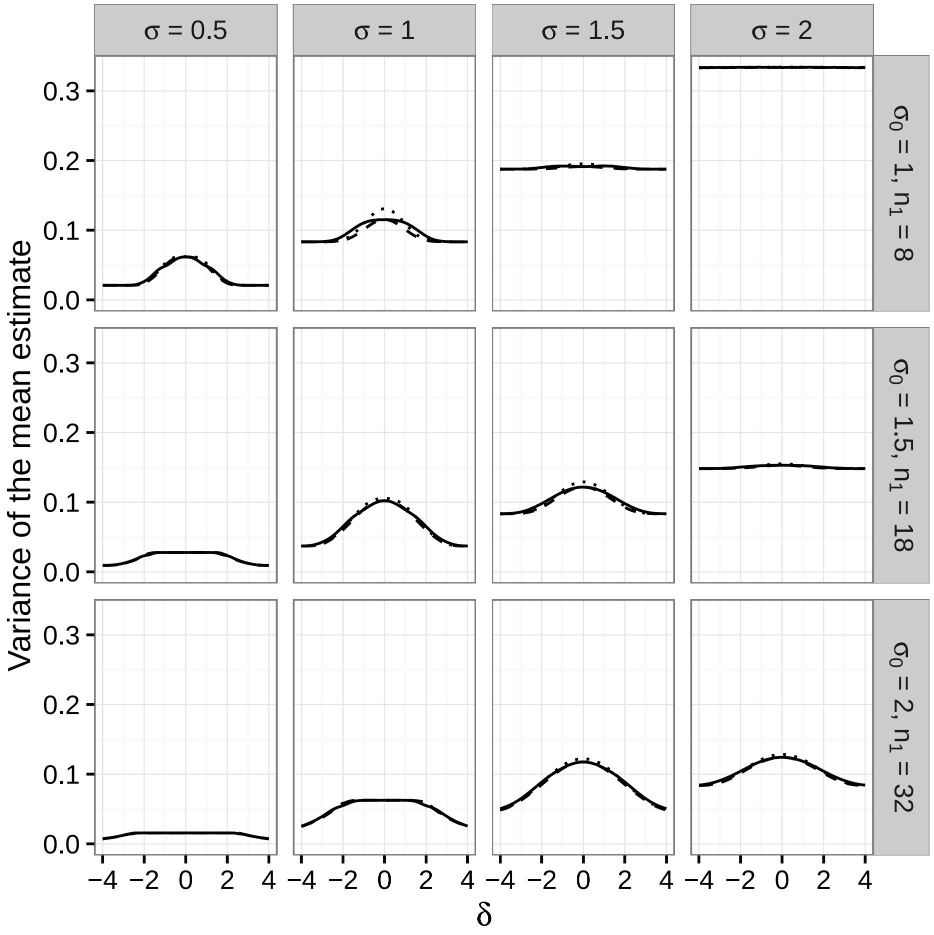

Figure 21: Adjusted sample size reassessment rule with a maximal second stage sample size limited to $2n_1$: actual variance of the mean estimate σ_e^2 (solid line), average of the estimated variances of the mean S_e^2 (dashed line), average of the variances σ_f^2 of the mean of fixed sample trials with the same sample sizes.

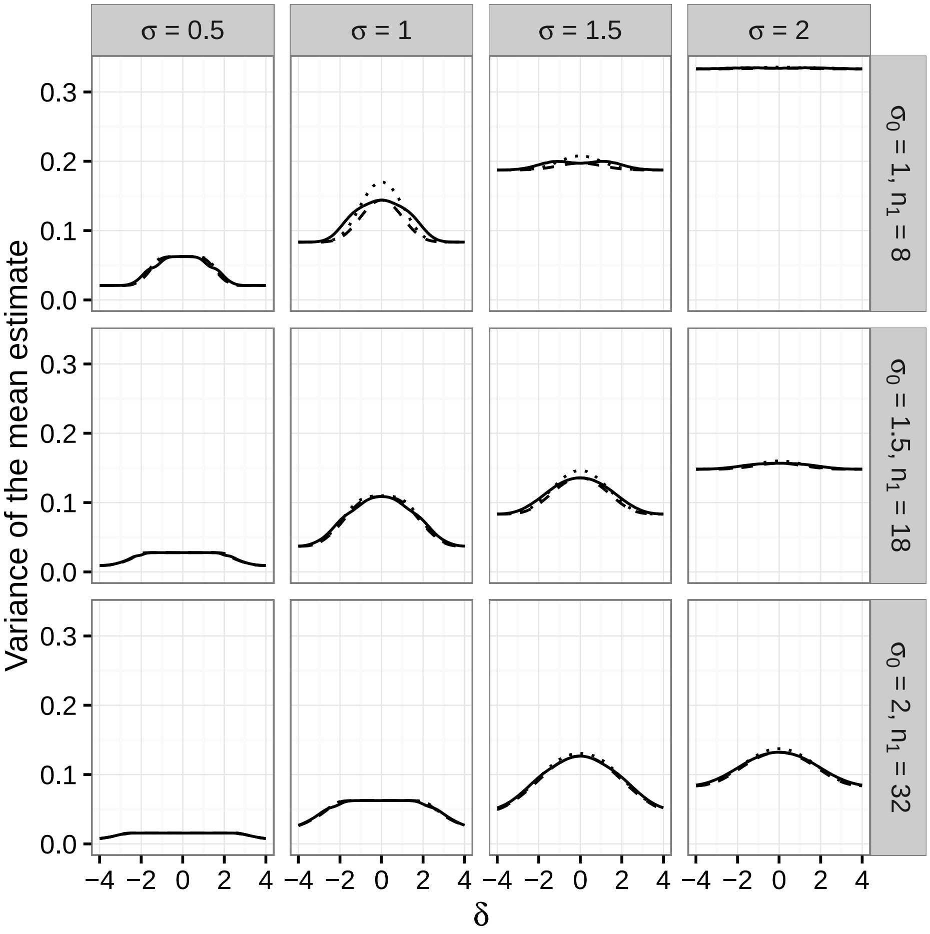

Figure 22: Variance of the effect estimates (i.e., square of the standard errors) for the unadjusted sample size reassessment rule with a maximal second stage sample size limited to $2n_1$: actual variance σ_e^2 of the mean estimate (solid line), average of the estimated variances S_e^2 of the mean (dashed line), average of the variance σ_f^2 of the mean of corresponding fixed sample trials (dotted line).

References

Kieser, M., and T. Friede. 2003. "Simple procedures for blinded sample size adjustment that do not affect the type I error rate." *Statistics in Medicine* 22: 3571—3581.

Lu, Kaifeng. 2016. "Distribution of the Two-Sample T-Test Statistic Following Blinded Sample Size Re-Estimation." *Pharmaceutical Statistics* 15. Wiley Online Library: 208–15.

Malsch, U., and M. Kieser. 2001. "Efficacy of Kava-Kava in the Treatment of Non-Psychotic Anxiety, Following Pretreatment with Benzodiazepines." *Psychopharmacology* 157 (3). Springer: 277–83.
